# Combination of Biodata Mining and Computational Modelling in Identification and Characterization of ORF1ab Polyprotein of SARS-CoV-2 Isolated from Oronasopharynx of an Iranian Patient

**DOI:** 10.1186/s12575-020-00121-9

**Published:** 2020-04-21

**Authors:** Reza Zolfaghari Emameh, Hassan Nosrati, Ramezan Ali Taheri

**Affiliations:** 1 Department of Energy and Environmental Biotechnology, National Institute of Genetic Engineering and Biotechnology (NIGEB), 14965/161, Tehran, Iran; 2 Department of Materials Engineering, Tarbiat Modares University, Tehran, Iran; 3 Nanobiotechnology Research Center, Baqiyatallah University of Medical Sciences, Tehran, Iran

**Keywords:** SARS-CoV-2, COVID-19, ORF1ab, nsp1, Biodata mining, Protein Modelling

## Abstract

**Background:**

Coronavirus disease 2019 (COVID-19) is an emerging zoonotic viral infection, which was started in Wuhan, China, in December 2019 and transmitted to other countries worldwide as a pandemic outbreak. Iran is one of the top ranked countries in the tables of COVID-19-infected and -mortality cases that make the Iranian patients as the potential targets for diversity of studies including epidemiology, biomedical, biodata, and viral proteins computational modelling studies.

**Results:**

In this study, we applied bioinformatic biodata mining methods to detect CDS and protein sequences of ORF1ab polyprotein of SARS-CoV-2 isolated from oronasopharynx of an Iranian patient. Then through the computational modelling and antigenicity prediction approaches, the identified polyprotein sequence was analyzed. The results revealed that the identified ORF1ab polyprotein belongs to a part of nonstructural protein 1 (nsp1) with the high antigenicity residues in a glycine-proline or hydrophobic amino acid rich domain.

**Conclusions:**

The results revealed that nsp1 as a virulence factor and crucial agent in spreading of the COVID-19 among the society can be a potential target for the future epidemiology, drug, and vaccine studies.

## Introduction

Coronaviruses (CoVs) are positive strand RNA viruses belong to the order of Nidovirales and three families including *Arteriviridae, Coronaviridae,* and *Roniviridae* [1]. Based on the genetic studies, CoVs are classified to into four genera including alpha, beta, gamma, and delta CoVs. The diameter of CoVs is between 80 to 120 nm and their shape is spherical. The spike projections of these virions give the appearance of solar corona to the CoVs. The main structural proteins of CoVs are envelope (E), membrane (M), nucleocapsid (N), and spike (S). The S proteins comprise N-linked signal peptide to be transferred to endoplasmic reticulum (ER) and consequently glycosylated in ER [2]. The homotrimeric structure of S glycoproteins on the surface of the CoVs mediate the attachment of virions to the cell receptors [3]. The size of positive-sense RNA genome of CoVs is between 26.2 and 31.7 kb. The RNA genome composes of six to ten open reading frames (ORFs). ORF1a as the longest part of the RNA encodes for the replicases and ORF1b expresses for two large polyproteins including pp1a and pp1ab comprising about 4000 and 7000 amino acids. The expression of pp1ab polyprotein is essential for programmed ribosomal frame shifting signal by bridging between ORF1a and ORF1ab [4]. In the CoVs, the frameshifting signal is led to the expression of a RNA-dependent RNA polymerase (RdRP), which is required for the coronavirus replication [5]. The polyproteins of CoVs are cleaved by virus-encoded cysteine proteinases comprise papain- and chymotrypsin-like proteases into 16 nonstructural proteins (nsp) including the expression of nsp1 to nsp11 by ORF1a and encoding nsp12 to nsp16 by ORF1b [6]. The nsp3, nsp4, and nsp6 contain hydrophobic transmembrane domains, which are considered as the anchor sites of pp1a and pp1ab polyproteins to membranes during the first step of formation of replication-transcription complexes (RTC). Further study defined that two out of three hydrophobic domains in nsp3 and six out of seven hydrophobic domains in nsp6 span the membrane, while four hydrophobic domains in nsp4 span to lipid bilayer [7]. On the other hand, ORF1b-encoded nsps including nsp12 has the RdRP activity, nsp13 has the helicase activity, nsp14 has the 3′ to 5′ exonuclease and RNA cap N7-guanine methyltransferase and activities for proofreading in association with nsp7/nsp8/nsp12 complex, and nsp15 has the endoribonuclease activity. The nsp16 has the methyltransferase activity, which in combination with helicase/triphosphatase, nsp13, and 2′O-MTase, a replication-transcription machinery is constituted to enable the CoVs in the RNA synthesis and processing steps [8].

CoVs cause zoonotic lethal human respiratory infections [9]. Severe Acute Respiratory Syndrome Coronavirus (SARS-CoV) was the causative agent of 2002–2003 outbreak that occurred in the Guangdong Province of China with mortality rate of 9% and 774 total deaths [10]. It is accepted that SARS-CoV was originated in Chinese bats that contain SARS-related CoVs with angiotensin converting enzyme 2 (ACE2) as the same host receptor, although the population working in the wet animal markets were the seropositive cases. In 2012, the CoVs were mutated to Middle East Respiratory Syndrome Coronavirus (MERS-CoV) or camel flu and obtained the human-to-human capability from the camel origin with mortality rate of 40% and 333 total deaths. The host cell receptor for MERS-CoV is Dipeptidyl peptidase 4 (DPP4), which is present in some other animal cells including bats, camels, horses, and rabbits [11, 12]. Up to 2019, the positive cases of MERS-CoV infection were 2374 and 823 total deaths from 27 countries [13]. Since the mouse model doesn’t express the DPP4 cell receptor, the vaccine studies against the MERS-CoV infection were focused on other vaccine model animals including *Macaca mulatta* (Rhesus macaques) [14, 15], *Callithrix jacchus* (common marmoset) [15–17], *Camelus dromedarius* (Dromedary camels) [18], hDPP4-transduced mice [19], transgenic mice expressing hDPP4 globally [20], hDPP4-humanized transgenic mice [21], CRISPR/Cas9-engineered mice [22], and hDPP4-knockin mice using CRISPR/Cas9 [23]. Since the big animals are not economic and easy handling, it is preferred that the smaller model animals with available testing vaccine efficiency methods to be applied in the MERS-CoV vaccine studies [24]. In addition, some potential vaccine candidates were produced against MERS-CoV infection using viral vectors including recombinant human adenovirus encoding for S protein [25–27], recombinant chimpanzee adenovirus encoding for S protein [28, 29], modified vaccinia virus Ankara encoding for S protein [29–31] and N protein [32], recombinant human adenovirus encoding for S protein with nanoparticle [33], DNA vaccine encoding for S protein [34–36], subunit vaccines for S protein, receptor binding domain of S protein, and recombinant N-terminal domain [37–48], virus-like particles encoding for S protein with nanoparticles [49–53], nanoparticles with ferritin displaying receptor-binding domain of S protein [54], inactivated whole- MERS-CoV [55–57], and live-attenuated MERS-CoV [58–62].

The phylogenetic studies and sequence analyses of SARS-CoV-2 and some SARS-related CoVs revealed that all use ACE2 as the host cell receptor [63]. Evolutionary, human SARS-CoVs and bat SARS-CoVs such as LYRa11, Rs3367, Rf1, Cp and Rp3 share a common ancestor, while SARS-CoV and MERS-CoV are distantly related to each other [64, 65]. Receptor-binding domain (RBD) of S protein, which is responsible for binding to ACE2 of cell host receptor, is considered as the major part evolving in the beta CoVs so 29 unique RBDs were phylogenetically identified in three distinct clades [66].

Based on the outbreak of SARS-CoV-2 as a novel member of CoVs in December 2019 in Wuhan, China, the causative agent of coronavirus disease 2019 (COVID-19), severity of symptoms, high human-to-human transmission rate, pandemic epidemiological situation, and high mortality rate (> 2,000,000 infected cases and > 120,000 deaths worldwide till mid-April 2020) [67], it is an urge to study SARS-CoV-2 in all aspects to discover potential pharmaceutical and vaccine candidates against COVID-19.

In this study, we evaluated the partial DNA sequence and the encoded ORF1ab polyprotein isolated from the oronasopharynx of an Iranian patient through combination of biodata mining and computational modelling methods to identify whether a potential domain is available for the stimulation of human immune system to consider it as a potential target of drug and vaccine studies.

## Results

### Sequence Analysis

**Fig. 1:**
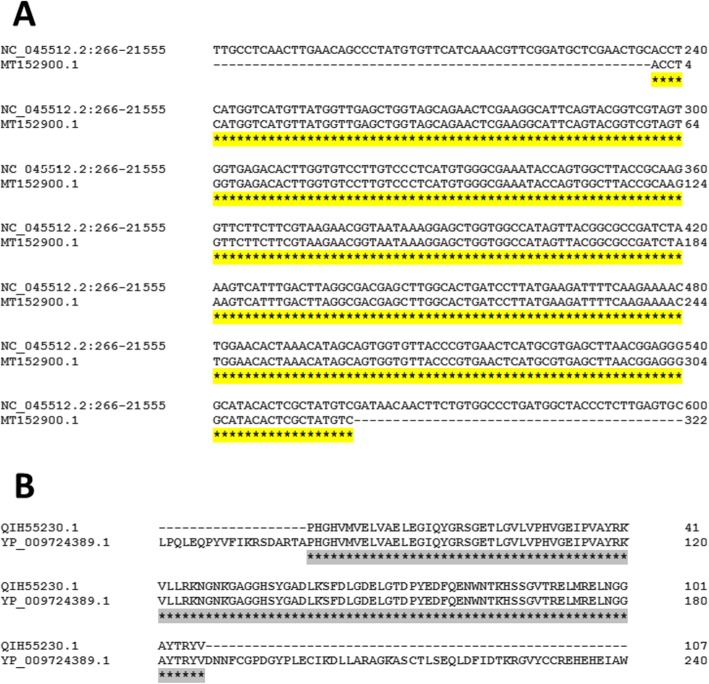
Multiple sequence alignment (MSA) analysis of ORF1ab polyprotein sequence. **a** MSA analysis of the Iranian CDS with the CDS query from Wuhan, China (yellow highlight); **b** MSA analysis of the Iranian partial ORF1ab polyprotein sequence with the query protein sequence from Wuhan, China (grey highlight). Both MSA evaluations show 100% identity

The multiple sequence alignment (MSA) analysis of the coding sequence (CDS) from the Iranian patient (Accession Number: MT152900) and the Chinese CDS query (Accession Number: NC_045512.2) revealed that the Iranian CDS sequence was located between nucleotides 237 and 558 (total length: 322 bases) of the Chinese CDS query with 100% sequence identity (Fig. 1a). In addition, the MSA analysis of the partial ORF1ab polyprotein from the Iranian patient (Accession Number: QIH55230) and the query protein sequence from Wuhan, China (Accession Number: YP_009724389.1) revealed that the Iranian protein sequence was a part of ORF1ab polyprotein from Wuhan, China (Accession Number: YP_009724389.1) with 100% sequence identity (Fig. 1b) (The complete information related to the Fig. 1a and b has been presented in the supplementary material 1).

### Protein Modelling

**Fig. 2:**
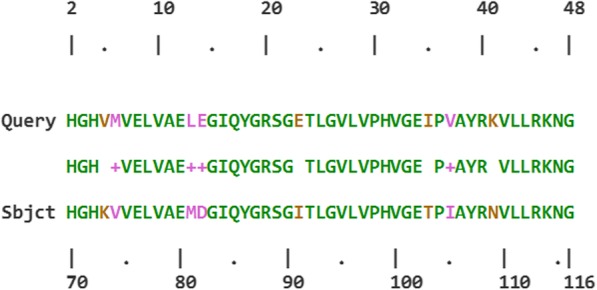
BLAST homology search analysis of the partial ORF1ab polyprotein sequence. The homology analysis defined that 39 out of 47 amino acids of the Iranian subject protein sequence are similar to the query protein sequence (NMR entry ID: 2GDT), which belonged to nsp1 from SARS-CoV

The protein modelling of the partial ORF1ab polyprotein sequence from the Iranian patient (Accession Number: QIH55230) in the RCSB PDB Protein Data Bank revealed that a 47-amino acid sequence of NMR entry ID: 2GDT belonged to nsp1 from the SARS-CoV with *E*-value: 4.20992E-16 and 83% identity, was the most similar model for the partial ORF1ab polyprotein sequence from the Iranian patient (Accession Number: QIH55230) (Fig. 2).

**Fig. 3:**
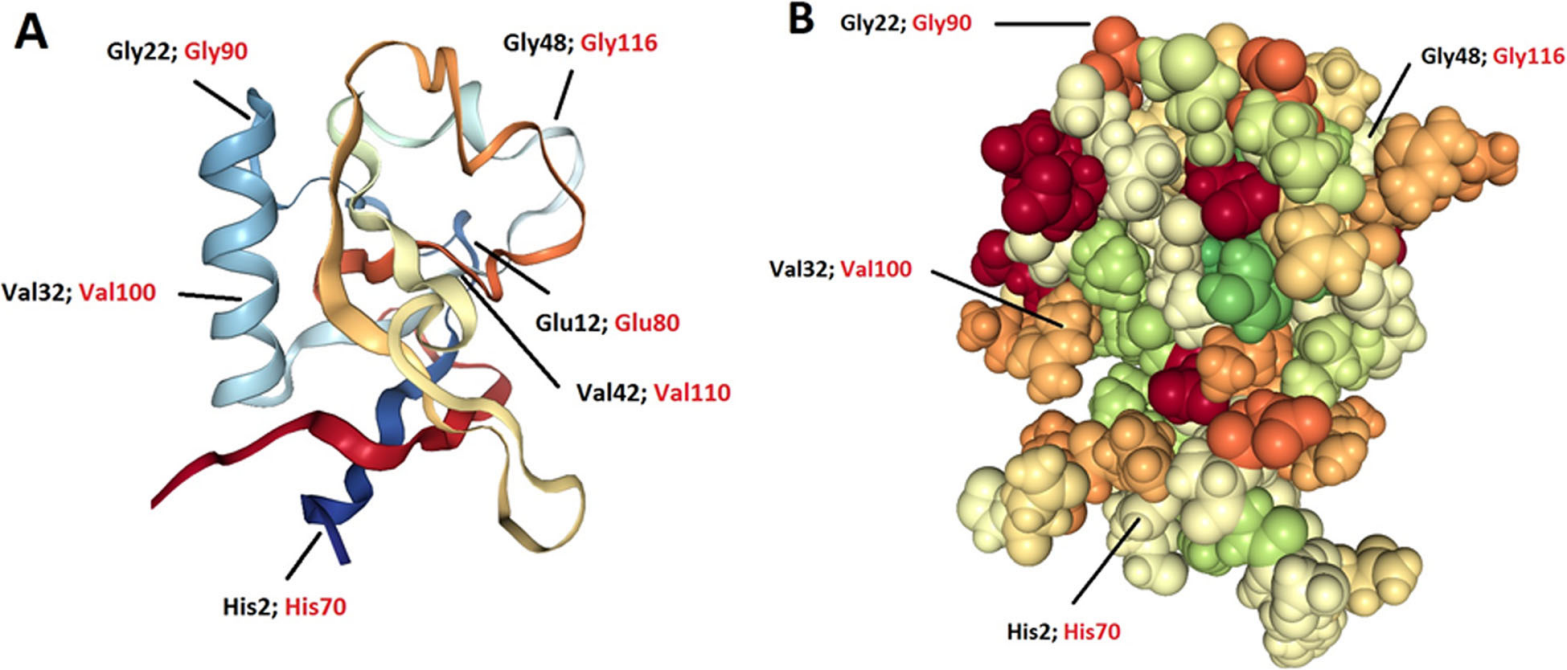
Protein Modelling of the partial ORF1ab polyprotein sequence. NGL (WebGL) viewer visualized the NMR structure of entry ID: 2GDT to (**a**) cartoon-rainbow style and (**b**) spacefill-hydrophobicity style. Both models show that partial ORF1ab polyprotein from the Iranian patient and nsp1 from SARS-CoV have highly similar structures. The black amino acids are from the query and the red amino acids are from the subject protein sequences

The visualization of the partial ORF1ab polyprotein sequence from the Iranian patient (Accession Number: QIH55230) by the NGL (WebGL) viewer revealed that the 3D model of the subject protein sequence has a considerable overlap with the query sequence. This 3D model overlap demonstrated that the partial ORF1ab polyprotein sequence from the Iranian patient (Accession Number: QIH55230) has a protein structure with close similarity to the nsp1 from SARS-CoV (Fig. 3).

### Antigenicity Prediction

The antigenicity prediction of partial ORF1ab polyprotein sequence from the Iranian patient (Accession Number: QIH55230) defined three antigenic domains including 22-, 13-, and 7-amino acids sequences. The most antigenic domain was the 22-amino acids domain located between Thr92 and Arg113 as the most antigenic domain. In the first ranked antigenic domain, 13 out of 22 amino acids were hydrophobic (Table 1).

**Table 1:**
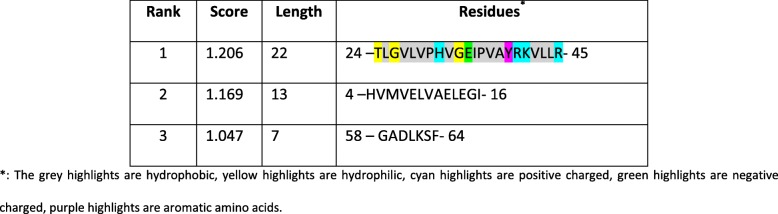
Antigenicity prediction of the partial ORF1ab polyprotein sequence

**Fig. 4:**
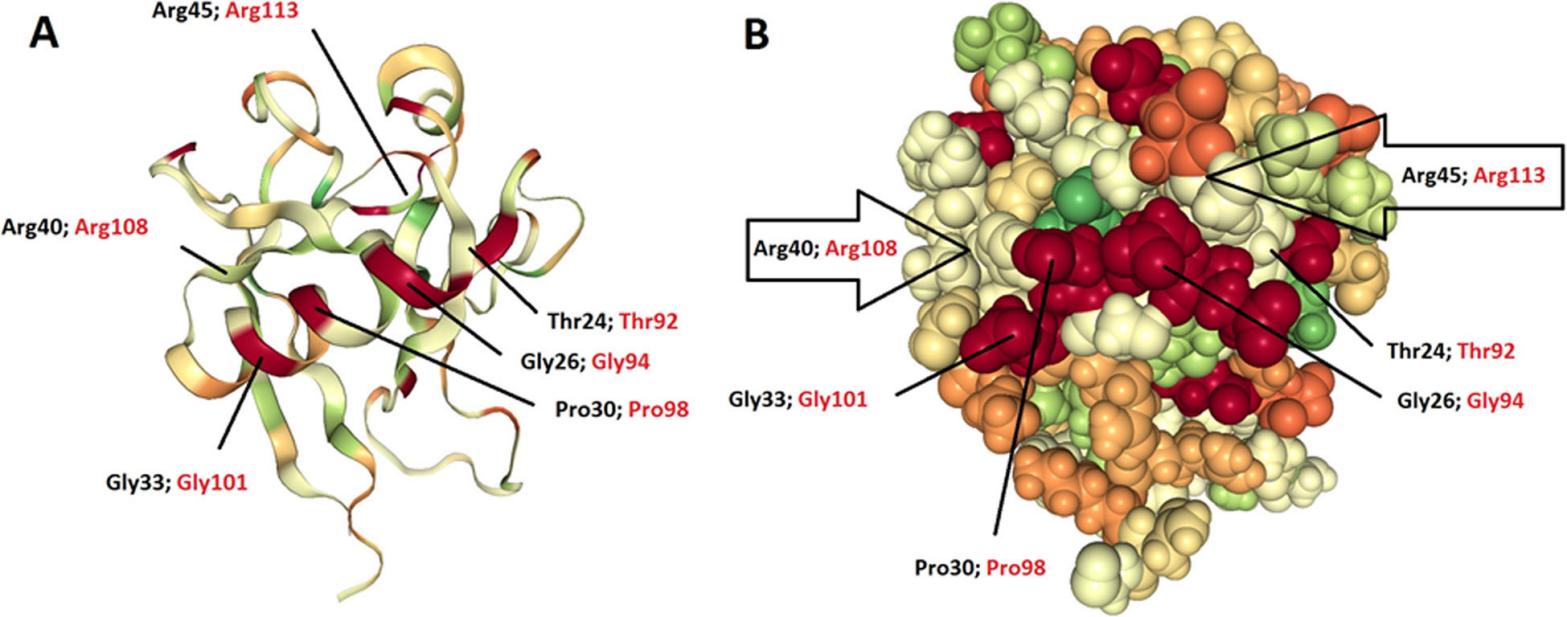
Amino acids locations of the most antigenic domain of the partial ORF1ab polyprotein sequence. **a** Cartoon-rainbow style and **b** spacefill-hydrophobicity style show the location of Gly94, Pro98, and Gly101 on the surface of the subject protein, which are accessible to the human immune system. The black amino acids are from the query and the red amino acids are from the subject protein sequences. The arrows show the buried amino acids

Further evaluation of the constituent amino acids of first ranked antigenic domain defined that the hydrophobic and hydrophilic amino acids were located at the surface of the subject protein and consequently access of human immune system, while other amino acids were buried and out of human immune system (Fig. 4).

## Discussion

SARS-CoV-2 infected many people from several cities of Iran since February 2020, which some studies are performing on different aspects of COVID-19.

The MSA analysis of the CDS and partial ORF1ab polyprotein sequence from the Iranian patient revealed that both CDS and partial ORF1ab polyprotein sequences of the Iranian sample were 100% identical to the query CDS sequence from Wuhan, China (Accession Number: NC_045512.2) and the query protein sequence from Wuhan, China (Accession Number: YP_009724389.1), respectively. These identities demonstrated that COVID-19 in Iran had the Wuhan origin of China, which was transmitted by human-to-human epidemiological pattern following a pandemic outbreak in other Asian Southeast countries such as Hong Kong, Japan and South Korea [68]. The protein modelling of the partial ORF1ab polyprotein sequence from the Iranian patient and detecting the NMR structure with PDB entry ID: 2GDT approved that the subject protein sequence from the Iranian patient is a part of nsp1 from SARS-CoV-2, which was 83% identical to the nsp1 from SARS-CoV. As it was identified, nsp1 is encoded by ORF1a and is highly conserved, crucial to the virus replication, survival in the society and spread among susceptible populations, and can be a potential virulence factor in COVID-19 through accelerating the cellular RNA degradation and consequently blocking the human immune response [69]. Since the BLASTP *E*-value scores for nsp1 from various isolates of SARS-CoV showed high percentage of identity, it was highly possible that the analyzed protein sequence was nsp1 and the Iranian patient had been affected by the virulence effect of identified nsp1 of SARS-CoV-2. The antigenicity prediction of the partial ORF1ab polyprotein or nsp1 sequence from the Iranian patient defined that firstly hydrophobic and secondly hydrophilic amino acids of the first ranked antigenic domain of partial nsp1 of the patient displayed higher antigenic properties with accessibility to the human immune system such as Gly94, Pro98, and Gly101. The previous studies showed that diversity of pathogenic and venomous living organisms produce glycine-proline rich antigens in the secretions or venoms [70–73]. Furthermore, glycine, proline, as well as hydrophobic amino acids were exposed on the surface of partial nsp1 of SARS-CoV-2 to play as a part of a virulence factor (nsp1) and stimulate the human immune system [74, 75].

## Conclusions

Although the identified protein sequence from an Iranian patient was a part of nsp1 from SARS-CoV-2 and could be a virulence and survival factor in the spreading of the COVID-19 among the population, there are some other potentials in nsp1 to make it attractive for future therapeutic and preventive strategies in pharmaceutical and vaccine manufacturers. Based on the highly conserved sequence of nsp1 among the isolates of SARS-CoVs, it can be an appropriate candidate in the molecular epidemiology of COVID-19 in the pandemic outbreaks.

## Methods

### Sequence Analysis

To obtain the data for DNA sequences of SARS-CoV-2 from Iran, we used NCBI Virus database (https://www.ncbi.nlm.nih.gov/labs/virus/vssi/#/) [76]. The accession number of MT152900 was selected that was related to the nucleotide sequence with 322 b in length isolated from the oronasopharynx of an Iranian patient annotated on the NCBI Virus database on 2020-02-26. The accession number MT152900 was annotated on the NCBI Virus database with the following details: Severe acute respiratory syndrome coronavirus 2 isolate SARS-CoV-2/MHKN-1/human/2020/IRN ORF1ab polyprotein (orf1ab) gene, partial cds (Karbalaie Niya,M.H., et al.). The accession number of the relevant protein sequence is QIH55230, which was nominated as the partial ORF1ab polyprotein with 107 amino acids in length.

The CDS sequence of the Iranian patient (Accession Number: MT152900) and the query CDS sequence from Wuhan, China (Accession Number: NC_045512.2) were compared using MSA and Clustal Omega algorithm (https://www.ebi.ac.uk/Tools/msa/clustalo/) [77]. In addition, the partial ORF1ab polyprotein from the Iranian patient (Accession Number: QIH55230) and the query protein sequence from Wuhan, China (Accession Number: YP_009724389.1) were compared using MSA and Clustal Omega algorithm as well.

### Protein Modelling

The partial ORF1ab polyprotein sequence from the Iranian patient (Accession Number: QIH55230) was searched in the RCSB PDB Protein Data Bank (https://www.rcsb.org/) [78]. A Basic Local Alignment Search Tool (BLAST) was employed by the Data Bank to identify the most identical crystalized protein structure to the subject protein sequence. Then, the NMR structure for the most identical crystalized protein structure would be visualized by NGL (WebGL) viewer [79].

### Antigenicity Prediction

The antigenicity prediction of the partial ORF1ab polyprotein sequence from the Iranian patient (Accession Number: QIH55230) was performed using EMBOSS antigenic explorer (https://www.bioinformatics.nl/cgi-bin/emboss/antigenic) [80]. This web tool predicts the potentially regions of the subject protein sequence through application of Kolaskar and Tongaonkar method on the hydrophobic residues of a protein domain. In addition, the amino acids location of the most antigenic domain of the subject protein was evaluated using NGL (WebGL) viewer within RCSB PDB Protein Data Bank.

## Supplementary information

**Additional file 1.**

## Data Availability

All data analyzed in this study were prepared from databases including NCBI Virus, RCSB PDB Protein Data Bank, and EMBOSS antigenic explorer and were included in this article.

## Abbreviations

ACE2: Angiotensin converting enzyme 2
BLAST: Basic Local Alignment Search Tool
CDS: Coding sequence
COVID-19: Coronavirus disease 2019
DPP4: Dipeptidyl peptidase 4
E: Envelope
ER: Endoplasmic reticulum
M: Membrane
MERS-CoV: Middle East Respiratory Syndrome Coronavirus
MSA: Multiple sequence alignment
N: Nucleocapsid
NCBI: National Center for Biotechnology Information
NMR: Nuclear magnetic resonance
nsp: Nonstructural protein
ORF: Open reading frame
PDB: Protein data bank
RBD: Receptor-binding domain
RdRP: RNA-dependent RNA polymerase
RNA: Ribonucleic acid
RTC: Replication-transcription complexes
S: Spike
SARS-CoV-2: Severe Acute Respiratory Syndrome Coronavirus 2
