# Supplementary material for: Combination of Biodata Mining and Computational Modelling in Identification and Characterization of ORF1ab Polyprotein of SARS-CoV-2 Isolated from Oronasopharynx of an Iranian Patient
Source: Biol Proced Online. 2020 Apr 21;22:8. doi: 10.1186/s12575-020-00121-9 (PMC7171442; doi:10.1186/s12575-020-00121-9)
Supplement: Supplementary file 1 — Additional file 1. [file 12575_2020_121_MOESM1_ESM.docx]

**A. MSA between the full ORF1ab CDS from Wuhan, China, and partial ORF1ab CDS from Iran**

CLUSTAL O(1.2.4) multiple sequence alignment

NC_045512.2:266-21555 ATGGAGAGCCTTGTCCCTGGTTTCAACGAGAAAACACACGTCCAACTCAGTTTGCCTGTT 60

MT152900.1 ------------------------------------------------------------ 0

NC_045512.2:266-21555 TTACAGGTTCGCGACGTGCTCGTACGTGGCTTTGGAGACTCCGTGGAGGAGGTCTTATCA 120

MT152900.1 ------------------------------------------------------------ 0

NC_045512.2:266-21555 GAGGCACGTCAACATCTTAAAGATGGCACTTGTGGCTTAGTAGAAGTTGAAAAAGGCGTT 180

MT152900.1 ------------------------------------------------------------ 0

NC_045512.2:266-21555 TTGCCTCAACTTGAACAGCCCTATGTGTTCATCAAACGTTCGGATGCTCGAACTGCACCT 240

MT152900.1 --------------------------------------------------------ACCT 4

****

NC_045512.2:266-21555 CATGGTCATGTTATGGTTGAGCTGGTAGCAGAACTCGAAGGCATTCAGTACGGTCGTAGT 300

MT152900.1 CATGGTCATGTTATGGTTGAGCTGGTAGCAGAACTCGAAGGCATTCAGTACGGTCGTAGT 64

************************************************************

NC_045512.2:266-21555 GGTGAGACACTTGGTGTCCTTGTCCCTCATGTGGGCGAAATACCAGTGGCTTACCGCAAG 360

MT152900.1 GGTGAGACACTTGGTGTCCTTGTCCCTCATGTGGGCGAAATACCAGTGGCTTACCGCAAG 124

************************************************************

NC_045512.2:266-21555 GTTCTTCTTCGTAAGAACGGTAATAAAGGAGCTGGTGGCCATAGTTACGGCGCCGATCTA 420

MT152900.1 GTTCTTCTTCGTAAGAACGGTAATAAAGGAGCTGGTGGCCATAGTTACGGCGCCGATCTA 184

************************************************************

NC_045512.2:266-21555 AAGTCATTTGACTTAGGCGACGAGCTTGGCACTGATCCTTATGAAGATTTTCAAGAAAAC 480

MT152900.1 AAGTCATTTGACTTAGGCGACGAGCTTGGCACTGATCCTTATGAAGATTTTCAAGAAAAC 244

************************************************************

NC_045512.2:266-21555 TGGAACACTAAACATAGCAGTGGTGTTACCCGTGAACTCATGCGTGAGCTTAACGGAGGG 540

MT152900.1 TGGAACACTAAACATAGCAGTGGTGTTACCCGTGAACTCATGCGTGAGCTTAACGGAGGG 304

************************************************************

NC_045512.2:266-21555 GCATACACTCGCTATGTCGATAACAACTTCTGTGGCCCTGATGGCTACCCTCTTGAGTGC 600

MT152900.1 GCATACACTCGCTATGTC------------------------------------------ 322

******************

NC_045512.2:266-21555 ATTAAAGACCTTCTAGCACGTGCTGGTAAAGCTTCATGCACTTTGTCCGAACAACTGGAC 660

MT152900.1 ------------------------------------------------------------ 322

NC_045512.2:266-21555 TTTATTGACACTAAGAGGGGTGTATACTGCTGCCGTGAACATGAGCATGAAATTGCTTGG 720

MT152900.1 ------------------------------------------------------------ 322

NC_045512.2:266-21555 TACACGGAACGTTCTGAAAAGAGCTATGAATTGCAGACACCTTTTGAAATTAAATTGGCA 780

MT152900.1 ------------------------------------------------------------ 322

NC_045512.2:266-21555 AAGAAATTTGACACCTTCAATGGGGAATGTCCAAATTTTGTATTTCCCTTAAATTCCATA 840

MT152900.1 ------------------------------------------------------------ 322

NC_045512.2:266-21555 ATCAAGACTATTCAACCAAGGGTTGAAAAGAAAAAGCTTGATGGCTTTATGGGTAGAATT 900

MT152900.1 ------------------------------------------------------------ 322

NC_045512.2:266-21555 CGATCTGTCTATCCAGTTGCGTCACCAAATGAATGCAACCAAATGTGCCTTTCAACTCTC 960

MT152900.1 ------------------------------------------------------------ 322

NC_045512.2:266-21555 ATGAAGTGTGATCATTGTGGTGAAACTTCATGGCAGACGGGCGATTTTGTTAAAGCCACT 1020

MT152900.1 ------------------------------------------------------------ 322

NC_045512.2:266-21555 TGCGAATTTTGTGGCACTGAGAATTTGACTAAAGAAGGTGCCACTACTTGTGGTTACTTA 1080

MT152900.1 ------------------------------------------------------------ 322

NC_045512.2:266-21555 CCCCAAAATGCTGTTGTTAAAATTTATTGTCCAGCATGTCACAATTCAGAAGTAGGACCT 1140

MT152900.1 ------------------------------------------------------------ 322

NC_045512.2:266-21555 GAGCATAGTCTTGCCGAATACCATAATGAATCTGGCTTGAAAACCATTCTTCGTAAGGGT 1200

MT152900.1 ------------------------------------------------------------ 322

NC_045512.2:266-21555 GGTCGCACTATTGCCTTTGGAGGCTGTGTGTTCTCTTATGTTGGTTGCCATAACAAGTGT 1260

MT152900.1 ------------------------------------------------------------ 322

NC_045512.2:266-21555 GCCTATTGGGTTCCACGTGCTAGCGCTAACATAGGTTGTAACCATACAGGTGTTGTTGGA 1320

MT152900.1 ------------------------------------------------------------ 322

NC_045512.2:266-21555 GAAGGTTCCGAAGGTCTTAATGACAACCTTCTTGAAATACTCCAAAAAGAGAAAGTCAAC 1380

MT152900.1 ------------------------------------------------------------ 322

NC_045512.2:266-21555 ATCAATATTGTTGGTGACTTTAAACTTAATGAAGAGATCGCCATTATTTTGGCATCTTTT 1440

MT152900.1 ------------------------------------------------------------ 322

NC_045512.2:266-21555 TCTGCTTCCACAAGTGCTTTTGTGGAAACTGTGAAAGGTTTGGATTATAAAGCATTCAAA 1500

MT152900.1 ------------------------------------------------------------ 322

NC_045512.2:266-21555 CAAATTGTTGAATCCTGTGGTAATTTTAAAGTTACAAAAGGAAAAGCTAAAAAAGGTGCC 1560

MT152900.1 ------------------------------------------------------------ 322

NC_045512.2:266-21555 TGGAATATTGGTGAACAGAAATCAATACTGAGTCCTCTTTATGCATTTGCATCAGAGGCT 1620

MT152900.1 ------------------------------------------------------------ 322

NC_045512.2:266-21555 GCTCGTGTTGTACGATCAATTTTCTCCCGCACTCTTGAAACTGCTCAAAATTCTGTGCGT 1680

MT152900.1 ------------------------------------------------------------ 322

NC_045512.2:266-21555 GTTTTACAGAAGGCCGCTATAACAATACTAGATGGAATTTCACAGTATTCACTGAGACTC 1740

MT152900.1 ------------------------------------------------------------ 322

NC_045512.2:266-21555 ATTGATGCTATGATGTTCACATCTGATTTGGCTACTAACAATCTAGTTGTAATGGCCTAC 1800

MT152900.1 ------------------------------------------------------------ 322

NC_045512.2:266-21555 ATTACAGGTGGTGTTGTTCAGTTGACTTCGCAGTGGCTAACTAACATCTTTGGCACTGTT 1860

MT152900.1 ------------------------------------------------------------ 322

NC_045512.2:266-21555 TATGAAAAACTCAAACCCGTCCTTGATTGGCTTGAAGAGAAGTTTAAGGAAGGTGTAGAG 1920

MT152900.1 ------------------------------------------------------------ 322

NC_045512.2:266-21555 TTTCTTAGAGACGGTTGGGAAATTGTTAAATTTATCTCAACCTGTGCTTGTGAAATTGTC 1980

MT152900.1 ------------------------------------------------------------ 322

NC_045512.2:266-21555 GGTGGACAAATTGTCACCTGTGCAAAGGAAATTAAGGAGAGTGTTCAGACATTCTTTAAG 2040

MT152900.1 ------------------------------------------------------------ 322

NC_045512.2:266-21555 CTTGTAAATAAATTTTTGGCTTTGTGTGCTGACTCTATCATTATTGGTGGAGCTAAACTT 2100

MT152900.1 ------------------------------------------------------------ 322

NC_045512.2:266-21555 AAAGCCTTGAATTTAGGTGAAACATTTGTCACGCACTCAAAGGGATTGTACAGAAAGTGT 2160

MT152900.1 ------------------------------------------------------------ 322

NC_045512.2:266-21555 GTTAAATCCAGAGAAGAAACTGGCCTACTCATGCCTCTAAAAGCCCCAAAAGAAATTATC 2220

MT152900.1 ------------------------------------------------------------ 322

NC_045512.2:266-21555 TTCTTAGAGGGAGAAACACTTCCCACAGAAGTGTTAACAGAGGAAGTTGTCTTGAAAACT 2280

MT152900.1 ------------------------------------------------------------ 322

NC_045512.2:266-21555 GGTGATTTACAACCATTAGAACAACCTACTAGTGAAGCTGTTGAAGCTCCATTGGTTGGT 2340

MT152900.1 ------------------------------------------------------------ 322

NC_045512.2:266-21555 ACACCAGTTTGTATTAACGGGCTTATGTTGCTCGAAATCAAAGACACAGAAAAGTACTGT 2400

MT152900.1 ------------------------------------------------------------ 322

NC_045512.2:266-21555 GCCCTTGCACCTAATATGATGGTAACAAACAATACCTTCACACTCAAAGGCGGTGCACCA 2460

MT152900.1 ------------------------------------------------------------ 322

NC_045512.2:266-21555 ACAAAGGTTACTTTTGGTGATGACACTGTGATAGAAGTGCAAGGTTACAAGAGTGTGAAT 2520

MT152900.1 ------------------------------------------------------------ 322

NC_045512.2:266-21555 ATCACTTTTGAACTTGATGAAAGGATTGATAAAGTACTTAATGAGAAGTGCTCTGCCTAT 2580

MT152900.1 ------------------------------------------------------------ 322

NC_045512.2:266-21555 ACAGTTGAACTCGGTACAGAAGTAAATGAGTTCGCCTGTGTTGTGGCAGATGCTGTCATA 2640

MT152900.1 ------------------------------------------------------------ 322

NC_045512.2:266-21555 AAAACTTTGCAACCAGTATCTGAATTACTTACACCACTGGGCATTGATTTAGATGAGTGG 2700

MT152900.1 ------------------------------------------------------------ 322

NC_045512.2:266-21555 AGTATGGCTACATACTACTTATTTGATGAGTCTGGTGAGTTTAAATTGGCTTCACATATG 2760

MT152900.1 ------------------------------------------------------------ 322

NC_045512.2:266-21555 TATTGTTCTTTCTACCCTCCAGATGAGGATGAAGAAGAAGGTGATTGTGAAGAAGAAGAG 2820

MT152900.1 ------------------------------------------------------------ 322

NC_045512.2:266-21555 TTTGAGCCATCAACTCAATATGAGTATGGTACTGAAGATGATTACCAAGGTAAACCTTTG 2880

MT152900.1 ------------------------------------------------------------ 322

NC_045512.2:266-21555 GAATTTGGTGCCACTTCTGCTGCTCTTCAACCTGAAGAAGAGCAAGAAGAAGATTGGTTA 2940

MT152900.1 ------------------------------------------------------------ 322

NC_045512.2:266-21555 GATGATGATAGTCAACAAACTGTTGGTCAACAAGACGGCAGTGAGGACAATCAGACAACT 3000

MT152900.1 ------------------------------------------------------------ 322

NC_045512.2:266-21555 ACTATTCAAACAATTGTTGAGGTTCAACCTCAATTAGAGATGGAACTTACACCAGTTGTT 3060

MT152900.1 ------------------------------------------------------------ 322

NC_045512.2:266-21555 CAGACTATTGAAGTGAATAGTTTTAGTGGTTATTTAAAACTTACTGACAATGTATACATT 3120

MT152900.1 ------------------------------------------------------------ 322

NC_045512.2:266-21555 AAAAATGCAGACATTGTGGAAGAAGCTAAAAAGGTAAAACCAACAGTGGTTGTTAATGCA 3180

MT152900.1 ------------------------------------------------------------ 322

NC_045512.2:266-21555 GCCAATGTTTACCTTAAACATGGAGGAGGTGTTGCAGGAGCCTTAAATAAGGCTACTAAC 3240

MT152900.1 ------------------------------------------------------------ 322

NC_045512.2:266-21555 AATGCCATGCAAGTTGAATCTGATGATTACATAGCTACTAATGGACCACTTAAAGTGGGT 3300

MT152900.1 ------------------------------------------------------------ 322

NC_045512.2:266-21555 GGTAGTTGTGTTTTAAGCGGACACAATCTTGCTAAACACTGTCTTCATGTTGTCGGCCCA 3360

MT152900.1 ------------------------------------------------------------ 322

NC_045512.2:266-21555 AATGTTAACAAAGGTGAAGACATTCAACTTCTTAAGAGTGCTTATGAAAATTTTAATCAG 3420

MT152900.1 ------------------------------------------------------------ 322

NC_045512.2:266-21555 CACGAAGTTCTACTTGCACCATTATTATCAGCTGGTATTTTTGGTGCTGACCCTATACAT 3480

MT152900.1 ------------------------------------------------------------ 322

NC_045512.2:266-21555 TCTTTAAGAGTTTGTGTAGATACTGTTCGCACAAATGTCTACTTAGCTGTCTTTGATAAA 3540

MT152900.1 ------------------------------------------------------------ 322

NC_045512.2:266-21555 AATCTCTATGACAAACTTGTTTCAAGCTTTTTGGAAATGAAGAGTGAAAAGCAAGTTGAA 3600

MT152900.1 ------------------------------------------------------------ 322

NC_045512.2:266-21555 CAAAAGATCGCTGAGATTCCTAAAGAGGAAGTTAAGCCATTTATAACTGAAAGTAAACCT 3660

MT152900.1 ------------------------------------------------------------ 322

NC_045512.2:266-21555 TCAGTTGAACAGAGAAAACAAGATGATAAGAAAATCAAAGCTTGTGTTGAAGAAGTTACA 3720

MT152900.1 ------------------------------------------------------------ 322

NC_045512.2:266-21555 ACAACTCTGGAAGAAACTAAGTTCCTCACAGAAAACTTGTTACTTTATATTGACATTAAT 3780

MT152900.1 ------------------------------------------------------------ 322

NC_045512.2:266-21555 GGCAATCTTCATCCAGATTCTGCCACTCTTGTTAGTGACATTGACATCACTTTCTTAAAG 3840

MT152900.1 ------------------------------------------------------------ 322

NC_045512.2:266-21555 AAAGATGCTCCATATATAGTGGGTGATGTTGTTCAAGAGGGTGTTTTAACTGCTGTGGTT 3900

MT152900.1 ------------------------------------------------------------ 322

NC_045512.2:266-21555 ATACCTACTAAAAAGGCTGGTGGCACTACTGAAATGCTAGCGAAAGCTTTGAGAAAAGTG 3960

MT152900.1 ------------------------------------------------------------ 322

NC_045512.2:266-21555 CCAACAGACAATTATATAACCACTTACCCGGGTCAGGGTTTAAATGGTTACACTGTAGAG 4020

MT152900.1 ------------------------------------------------------------ 322

NC_045512.2:266-21555 GAGGCAAAGACAGTGCTTAAAAAGTGTAAAAGTGCCTTTTACATTCTACCATCTATTATC 4080

MT152900.1 ------------------------------------------------------------ 322

NC_045512.2:266-21555 TCTAATGAGAAGCAAGAAATTCTTGGAACTGTTTCTTGGAATTTGCGAGAAATGCTTGCA 4140

MT152900.1 ------------------------------------------------------------ 322

NC_045512.2:266-21555 CATGCAGAAGAAACACGCAAATTAATGCCTGTCTGTGTGGAAACTAAAGCCATAGTTTCA 4200

MT152900.1 ------------------------------------------------------------ 322

NC_045512.2:266-21555 ACTATACAGCGTAAATATAAGGGTATTAAAATACAAGAGGGTGTGGTTGATTATGGTGCT 4260

MT152900.1 ------------------------------------------------------------ 322

NC_045512.2:266-21555 AGATTTTACTTTTACACCAGTAAAACAACTGTAGCGTCACTTATCAACACACTTAACGAT 4320

MT152900.1 ------------------------------------------------------------ 322

NC_045512.2:266-21555 CTAAATGAAACTCTTGTTACAATGCCACTTGGCTATGTAACACATGGCTTAAATTTGGAA 4380

MT152900.1 ------------------------------------------------------------ 322

NC_045512.2:266-21555 GAAGCTGCTCGGTATATGAGATCTCTCAAAGTGCCAGCTACAGTTTCTGTTTCTTCACCT 4440

MT152900.1 ------------------------------------------------------------ 322

NC_045512.2:266-21555 GATGCTGTTACAGCGTATAATGGTTATCTTACTTCTTCTTCTAAAACACCTGAAGAACAT 4500

MT152900.1 ------------------------------------------------------------ 322

NC_045512.2:266-21555 TTTATTGAAACCATCTCACTTGCTGGTTCCTATAAAGATTGGTCCTATTCTGGACAATCT 4560

MT152900.1 ------------------------------------------------------------ 322

NC_045512.2:266-21555 ACACAACTAGGTATAGAATTTCTTAAGAGAGGTGATAAAAGTGTATATTACACTAGTAAT 4620

MT152900.1 ------------------------------------------------------------ 322

NC_045512.2:266-21555 CCTACCACATTCCACCTAGATGGTGAAGTTATCACCTTTGACAATCTTAAGACACTTCTT 4680

MT152900.1 ------------------------------------------------------------ 322

NC_045512.2:266-21555 TCTTTGAGAGAAGTGAGGACTATTAAGGTGTTTACAACAGTAGACAACATTAACCTCCAC 4740

MT152900.1 ------------------------------------------------------------ 322

NC_045512.2:266-21555 ACGCAAGTTGTGGACATGTCAATGACATATGGACAACAGTTTGGTCCAACTTATTTGGAT 4800

MT152900.1 ------------------------------------------------------------ 322

NC_045512.2:266-21555 GGAGCTGATGTTACTAAAATAAAACCTCATAATTCACATGAAGGTAAAACATTTTATGTT 4860

MT152900.1 ------------------------------------------------------------ 322

NC_045512.2:266-21555 TTACCTAATGATGACACTCTACGTGTTGAGGCTTTTGAGTACTACCACACAACTGATCCT 4920

MT152900.1 ------------------------------------------------------------ 322

NC_045512.2:266-21555 AGTTTTCTGGGTAGGTACATGTCAGCATTAAATCACACTAAAAAGTGGAAATACCCACAA 4980

MT152900.1 ------------------------------------------------------------ 322

NC_045512.2:266-21555 GTTAATGGTTTAACTTCTATTAAATGGGCAGATAACAACTGTTATCTTGCCACTGCATTG 5040

MT152900.1 ------------------------------------------------------------ 322

NC_045512.2:266-21555 TTAACACTCCAACAAATAGAGTTGAAGTTTAATCCACCTGCTCTACAAGATGCTTATTAC 5100

MT152900.1 ------------------------------------------------------------ 322

NC_045512.2:266-21555 AGAGCAAGGGCTGGTGAAGCTGCTAACTTTTGTGCACTTATCTTAGCCTACTGTAATAAG 5160

MT152900.1 ------------------------------------------------------------ 322

NC_045512.2:266-21555 ACAGTAGGTGAGTTAGGTGATGTTAGAGAAACAATGAGTTACTTGTTTCAACATGCCAAT 5220

MT152900.1 ------------------------------------------------------------ 322

NC_045512.2:266-21555 TTAGATTCTTGCAAAAGAGTCTTGAACGTGGTGTGTAAAACTTGTGGACAACAGCAGACA 5280

MT152900.1 ------------------------------------------------------------ 322

NC_045512.2:266-21555 ACCCTTAAGGGTGTAGAAGCTGTTATGTACATGGGCACACTTTCTTATGAACAATTTAAG 5340

MT152900.1 ------------------------------------------------------------ 322

NC_045512.2:266-21555 AAAGGTGTTCAGATACCTTGTACGTGTGGTAAACAAGCTACAAAATATCTAGTACAACAG 5400

MT152900.1 ------------------------------------------------------------ 322

NC_045512.2:266-21555 GAGTCACCTTTTGTTATGATGTCAGCACCACCTGCTCAGTATGAACTTAAGCATGGTACA 5460

MT152900.1 ------------------------------------------------------------ 322

NC_045512.2:266-21555 TTTACTTGTGCTAGTGAGTACACTGGTAATTACCAGTGTGGTCACTATAAACATATAACT 5520

MT152900.1 ------------------------------------------------------------ 322

NC_045512.2:266-21555 TCTAAAGAAACTTTGTATTGCATAGACGGTGCTTTACTTACAAAGTCCTCAGAATACAAA 5580

MT152900.1 ------------------------------------------------------------ 322

NC_045512.2:266-21555 GGTCCTATTACGGATGTTTTCTACAAAGAAAACAGTTACACAACAACCATAAAACCAGTT 5640

MT152900.1 ------------------------------------------------------------ 322

NC_045512.2:266-21555 ACTTATAAATTGGATGGTGTTGTTTGTACAGAAATTGACCCTAAGTTGGACAATTATTAT 5700

MT152900.1 ------------------------------------------------------------ 322

NC_045512.2:266-21555 AAGAAAGACAATTCTTATTTCACAGAGCAACCAATTGATCTTGTACCAAACCAACCATAT 5760

MT152900.1 ------------------------------------------------------------ 322

NC_045512.2:266-21555 CCAAACGCAAGCTTCGATAATTTTAAGTTTGTATGTGATAATATCAAATTTGCTGATGAT 5820

MT152900.1 ------------------------------------------------------------ 322

NC_045512.2:266-21555 TTAAACCAGTTAACTGGTTATAAGAAACCTGCTTCAAGAGAGCTTAAAGTTACATTTTTC 5880

MT152900.1 ------------------------------------------------------------ 322

NC_045512.2:266-21555 CCTGACTTAAATGGTGATGTGGTGGCTATTGATTATAAACACTACACACCCTCTTTTAAG 5940

MT152900.1 ------------------------------------------------------------ 322

NC_045512.2:266-21555 AAAGGAGCTAAATTGTTACATAAACCTATTGTTTGGCATGTTAACAATGCAACTAATAAA 6000

MT152900.1 ------------------------------------------------------------ 322

NC_045512.2:266-21555 GCCACGTATAAACCAAATACCTGGTGTATACGTTGTCTTTGGAGCACAAAACCAGTTGAA 6060

MT152900.1 ------------------------------------------------------------ 322

NC_045512.2:266-21555 ACATCAAATTCGTTTGATGTACTGAAGTCAGAGGACGCGCAGGGAATGGATAATCTTGCC 6120

MT152900.1 ------------------------------------------------------------ 322

NC_045512.2:266-21555 TGCGAAGATCTAAAACCAGTCTCTGAAGAAGTAGTGGAAAATCCTACCATACAGAAAGAC 6180

MT152900.1 ------------------------------------------------------------ 322

NC_045512.2:266-21555 GTTCTTGAGTGTAATGTGAAAACTACCGAAGTTGTAGGAGACATTATACTTAAACCAGCA 6240

MT152900.1 ------------------------------------------------------------ 322

NC_045512.2:266-21555 AATAATAGTTTAAAAATTACAGAAGAGGTTGGCCACACAGATCTAATGGCTGCTTATGTA 6300

MT152900.1 ------------------------------------------------------------ 322

NC_045512.2:266-21555 GACAATTCTAGTCTTACTATTAAGAAACCTAATGAATTATCTAGAGTATTAGGTTTGAAA 6360

MT152900.1 ------------------------------------------------------------ 322

NC_045512.2:266-21555 ACCCTTGCTACTCATGGTTTAGCTGCTGTTAATAGTGTCCCTTGGGATACTATAGCTAAT 6420

MT152900.1 ------------------------------------------------------------ 322

NC_045512.2:266-21555 TATGCTAAGCCTTTTCTTAACAAAGTTGTTAGTACAACTACTAACATAGTTACACGGTGT 6480

MT152900.1 ------------------------------------------------------------ 322

NC_045512.2:266-21555 TTAAACCGTGTTTGTACTAATTATATGCCTTATTTCTTTACTTTATTGCTACAATTGTGT 6540

MT152900.1 ------------------------------------------------------------ 322

NC_045512.2:266-21555 ACTTTTACTAGAAGTACAAATTCTAGAATTAAAGCATCTATGCCGACTACTATAGCAAAG 6600

MT152900.1 ------------------------------------------------------------ 322

NC_045512.2:266-21555 AATACTGTTAAGAGTGTCGGTAAATTTTGTCTAGAGGCTTCATTTAATTATTTGAAGTCA 6660

MT152900.1 ------------------------------------------------------------ 322

NC_045512.2:266-21555 CCTAATTTTTCTAAACTGATAAATATTATAATTTGGTTTTTACTATTAAGTGTTTGCCTA 6720

MT152900.1 ------------------------------------------------------------ 322

NC_045512.2:266-21555 GGTTCTTTAATCTACTCAACCGCTGCTTTAGGTGTTTTAATGTCTAATTTAGGCATGCCT 6780

MT152900.1 ------------------------------------------------------------ 322

NC_045512.2:266-21555 TCTTACTGTACTGGTTACAGAGAAGGCTATTTGAACTCTACTAATGTCACTATTGCAACC 6840

MT152900.1 ------------------------------------------------------------ 322

NC_045512.2:266-21555 TACTGTACTGGTTCTATACCTTGTAGTGTTTGTCTTAGTGGTTTAGATTCTTTAGACACC 6900

MT152900.1 ------------------------------------------------------------ 322

NC_045512.2:266-21555 TATCCTTCTTTAGAAACTATACAAATTACCATTTCATCTTTTAAATGGGATTTAACTGCT 6960

MT152900.1 ------------------------------------------------------------ 322

NC_045512.2:266-21555 TTTGGCTTAGTTGCAGAGTGGTTTTTGGCATATATTCTTTTCACTAGGTTTTTCTATGTA 7020

MT152900.1 ------------------------------------------------------------ 322

NC_045512.2:266-21555 CTTGGATTGGCTGCAATCATGCAATTGTTTTTCAGCTATTTTGCAGTACATTTTATTAGT 7080

MT152900.1 ------------------------------------------------------------ 322

NC_045512.2:266-21555 AATTCTTGGCTTATGTGGTTAATAATTAATCTTGTACAAATGGCCCCGATTTCAGCTATG 7140

MT152900.1 ------------------------------------------------------------ 322

NC_045512.2:266-21555 GTTAGAATGTACATCTTCTTTGCATCATTTTATTATGTATGGAAAAGTTATGTGCATGTT 7200

MT152900.1 ------------------------------------------------------------ 322

NC_045512.2:266-21555 GTAGACGGTTGTAATTCATCAACTTGTATGATGTGTTACAAACGTAATAGAGCAACAAGA 7260

MT152900.1 ------------------------------------------------------------ 322

NC_045512.2:266-21555 GTCGAATGTACAACTATTGTTAATGGTGTTAGAAGGTCCTTTTATGTCTATGCTAATGGA 7320

MT152900.1 ------------------------------------------------------------ 322

NC_045512.2:266-21555 GGTAAAGGCTTTTGCAAACTACACAATTGGAATTGTGTTAATTGTGATACATTCTGTGCT 7380

MT152900.1 ------------------------------------------------------------ 322

NC_045512.2:266-21555 GGTAGTACATTTATTAGTGATGAAGTTGCGAGAGACTTGTCACTACAGTTTAAAAGACCA 7440

MT152900.1 ------------------------------------------------------------ 322

NC_045512.2:266-21555 ATAAATCCTACTGACCAGTCTTCTTACATCGTTGATAGTGTTACAGTGAAGAATGGTTCC 7500

MT152900.1 ------------------------------------------------------------ 322

NC_045512.2:266-21555 ATCCATCTTTACTTTGATAAAGCTGGTCAAAAGACTTATGAAAGACATTCTCTCTCTCAT 7560

MT152900.1 ------------------------------------------------------------ 322

NC_045512.2:266-21555 TTTGTTAACTTAGACAACCTGAGAGCTAATAACACTAAAGGTTCATTGCCTATTAATGTT 7620

MT152900.1 ------------------------------------------------------------ 322

NC_045512.2:266-21555 ATAGTTTTTGATGGTAAATCAAAATGTGAAGAATCATCTGCAAAATCAGCGTCTGTTTAC 7680

MT152900.1 ------------------------------------------------------------ 322

NC_045512.2:266-21555 TACAGTCAGCTTATGTGTCAACCTATACTGTTACTAGATCAGGCATTAGTGTCTGATGTT 7740

MT152900.1 ------------------------------------------------------------ 322

NC_045512.2:266-21555 GGTGATAGTGCGGAAGTTGCAGTTAAAATGTTTGATGCTTACGTTAATACGTTTTCATCA 7800

MT152900.1 ------------------------------------------------------------ 322

NC_045512.2:266-21555 ACTTTTAACGTACCAATGGAAAAACTCAAAACACTAGTTGCAACTGCAGAAGCTGAACTT 7860

MT152900.1 ------------------------------------------------------------ 322

NC_045512.2:266-21555 GCAAAGAATGTGTCCTTAGACAATGTCTTATCTACTTTTATTTCAGCAGCTCGGCAAGGG 7920

MT152900.1 ------------------------------------------------------------ 322

NC_045512.2:266-21555 TTTGTTGATTCAGATGTAGAAACTAAAGATGTTGTTGAATGTCTTAAATTGTCACATCAA 7980

MT152900.1 ------------------------------------------------------------ 322

NC_045512.2:266-21555 TCTGACATAGAAGTTACTGGCGATAGTTGTAATAACTATATGCTCACCTATAACAAAGTT 8040

MT152900.1 ------------------------------------------------------------ 322

NC_045512.2:266-21555 GAAAACATGACACCCCGTGACCTTGGTGCTTGTATTGACTGTAGTGCGCGTCATATTAAT 8100

MT152900.1 ------------------------------------------------------------ 322

NC_045512.2:266-21555 GCGCAGGTAGCAAAAAGTCACAACATTGCTTTGATATGGAACGTTAAAGATTTCATGTCA 8160

MT152900.1 ------------------------------------------------------------ 322

NC_045512.2:266-21555 TTGTCTGAACAACTACGAAAACAAATACGTAGTGCTGCTAAAAAGAATAACTTACCTTTT 8220

MT152900.1 ------------------------------------------------------------ 322

NC_045512.2:266-21555 AAGTTGACATGTGCAACTACTAGACAAGTTGTTAATGTTGTAACAACAAAGATAGCACTT 8280

MT152900.1 ------------------------------------------------------------ 322

NC_045512.2:266-21555 AAGGGTGGTAAAATTGTTAATAATTGGTTGAAGCAGTTAATTAAAGTTACACTTGTGTTC 8340

MT152900.1 ------------------------------------------------------------ 322

NC_045512.2:266-21555 CTTTTTGTTGCTGCTATTTTCTATTTAATAACACCTGTTCATGTCATGTCTAAACATACT 8400

MT152900.1 ------------------------------------------------------------ 322

NC_045512.2:266-21555 GACTTTTCAAGTGAAATCATAGGATACAAGGCTATTGATGGTGGTGTCACTCGTGACATA 8460

MT152900.1 ------------------------------------------------------------ 322

NC_045512.2:266-21555 GCATCTACAGATACTTGTTTTGCTAACAAACATGCTGATTTTGACACATGGTTTAGCCAG 8520

MT152900.1 ------------------------------------------------------------ 322

NC_045512.2:266-21555 CGTGGTGGTAGTTATACTAATGACAAAGCTTGCCCATTGATTGCTGCAGTCATAACAAGA 8580

MT152900.1 ------------------------------------------------------------ 322

NC_045512.2:266-21555 GAAGTGGGTTTTGTCGTGCCTGGTTTGCCTGGCACGATATTACGCACAACTAATGGTGAC 8640

MT152900.1 ------------------------------------------------------------ 322

NC_045512.2:266-21555 TTTTTGCATTTCTTACCTAGAGTTTTTAGTGCAGTTGGTAACATCTGTTACACACCATCA 8700

MT152900.1 ------------------------------------------------------------ 322

NC_045512.2:266-21555 AAACTTATAGAGTACACTGACTTTGCAACATCAGCTTGTGTTTTGGCTGCTGAATGTACA 8760

MT152900.1 ------------------------------------------------------------ 322

NC_045512.2:266-21555 ATTTTTAAAGATGCTTCTGGTAAGCCAGTACCATATTGTTATGATACCAATGTACTAGAA 8820

MT152900.1 ------------------------------------------------------------ 322

NC_045512.2:266-21555 GGTTCTGTTGCTTATGAAAGTTTACGCCCTGACACACGTTATGTGCTCATGGATGGCTCT 8880

MT152900.1 ------------------------------------------------------------ 322

NC_045512.2:266-21555 ATTATTCAATTTCCTAACACCTACCTTGAAGGTTCTGTTAGAGTGGTAACAACTTTTGAT 8940

MT152900.1 ------------------------------------------------------------ 322

NC_045512.2:266-21555 TCTGAGTACTGTAGGCACGGCACTTGTGAAAGATCAGAAGCTGGTGTTTGTGTATCTACT 9000

MT152900.1 ------------------------------------------------------------ 322

NC_045512.2:266-21555 AGTGGTAGATGGGTACTTAACAATGATTATTACAGATCTTTACCAGGAGTTTTCTGTGGT 9060

MT152900.1 ------------------------------------------------------------ 322

NC_045512.2:266-21555 GTAGATGCTGTAAATTTACTTACTAATATGTTTACACCACTAATTCAACCTATTGGTGCT 9120

MT152900.1 ------------------------------------------------------------ 322

NC_045512.2:266-21555 TTGGACATATCAGCATCTATAGTAGCTGGTGGTATTGTAGCTATCGTAGTAACATGCCTT 9180

MT152900.1 ------------------------------------------------------------ 322

NC_045512.2:266-21555 GCCTACTATTTTATGAGGTTTAGAAGAGCTTTTGGTGAATACAGTCATGTAGTTGCCTTT 9240

MT152900.1 ------------------------------------------------------------ 322

NC_045512.2:266-21555 AATACTTTACTATTCCTTATGTCATTCACTGTACTCTGTTTAACACCAGTTTACTCATTC 9300

MT152900.1 ------------------------------------------------------------ 322

NC_045512.2:266-21555 TTACCTGGTGTTTATTCTGTTATTTACTTGTACTTGACATTTTATCTTACTAATGATGTT 9360

MT152900.1 ------------------------------------------------------------ 322

NC_045512.2:266-21555 TCTTTTTTAGCACATATTCAGTGGATGGTTATGTTCACACCTTTAGTACCTTTCTGGATA 9420

MT152900.1 ------------------------------------------------------------ 322

NC_045512.2:266-21555 ACAATTGCTTATATCATTTGTATTTCCACAAAGCATTTCTATTGGTTCTTTAGTAATTAC 9480

MT152900.1 ------------------------------------------------------------ 322

NC_045512.2:266-21555 CTAAAGAGACGTGTAGTCTTTAATGGTGTTTCCTTTAGTACTTTTGAAGAAGCTGCGCTG 9540

MT152900.1 ------------------------------------------------------------ 322

NC_045512.2:266-21555 TGCACCTTTTTGTTAAATAAAGAAATGTATCTAAAGTTGCGTAGTGATGTGCTATTACCT 9600

MT152900.1 ------------------------------------------------------------ 322

NC_045512.2:266-21555 CTTACGCAATATAATAGATACTTAGCTCTTTATAATAAGTACAAGTATTTTAGTGGAGCA 9660

MT152900.1 ------------------------------------------------------------ 322

NC_045512.2:266-21555 ATGGATACAACTAGCTACAGAGAAGCTGCTTGTTGTCATCTCGCAAAGGCTCTCAATGAC 9720

MT152900.1 ------------------------------------------------------------ 322

NC_045512.2:266-21555 TTCAGTAACTCAGGTTCTGATGTTCTTTACCAACCACCACAAACCTCTATCACCTCAGCT 9780

MT152900.1 ------------------------------------------------------------ 322

NC_045512.2:266-21555 GTTTTGCAGAGTGGTTTTAGAAAAATGGCATTCCCATCTGGTAAAGTTGAGGGTTGTATG 9840

MT152900.1 ------------------------------------------------------------ 322

NC_045512.2:266-21555 GTACAAGTAACTTGTGGTACAACTACACTTAACGGTCTTTGGCTTGATGACGTAGTTTAC 9900

MT152900.1 ------------------------------------------------------------ 322

NC_045512.2:266-21555 TGTCCAAGACATGTGATCTGCACCTCTGAAGACATGCTTAACCCTAATTATGAAGATTTA 9960

MT152900.1 ------------------------------------------------------------ 322

NC_045512.2:266-21555 CTCATTCGTAAGTCTAATCATAATTTCTTGGTACAGGCTGGTAATGTTCAACTCAGGGTT 10020

MT152900.1 ------------------------------------------------------------ 322

NC_045512.2:266-21555 ATTGGACATTCTATGCAAAATTGTGTACTTAAGCTTAAGGTTGATACAGCCAATCCTAAG 10080

MT152900.1 ------------------------------------------------------------ 322

NC_045512.2:266-21555 ACACCTAAGTATAAGTTTGTTCGCATTCAACCAGGACAGACTTTTTCAGTGTTAGCTTGT 10140

MT152900.1 ------------------------------------------------------------ 322

NC_045512.2:266-21555 TACAATGGTTCACCATCTGGTGTTTACCAATGTGCTATGAGGCCCAATTTCACTATTAAG 10200

MT152900.1 ------------------------------------------------------------ 322

NC_045512.2:266-21555 GGTTCATTCCTTAATGGTTCATGTGGTAGTGTTGGTTTTAACATAGATTATGACTGTGTC 10260

MT152900.1 ------------------------------------------------------------ 322

NC_045512.2:266-21555 TCTTTTTGTTACATGCACCATATGGAATTACCAACTGGAGTTCATGCTGGCACAGACTTA 10320

MT152900.1 ------------------------------------------------------------ 322

NC_045512.2:266-21555 GAAGGTAACTTTTATGGACCTTTTGTTGACAGGCAAACAGCACAAGCAGCTGGTACGGAC 10380

MT152900.1 ------------------------------------------------------------ 322

NC_045512.2:266-21555 ACAACTATTACAGTTAATGTTTTAGCTTGGTTGTACGCTGCTGTTATAAATGGAGACAGG 10440

MT152900.1 ------------------------------------------------------------ 322

NC_045512.2:266-21555 TGGTTTCTCAATCGATTTACCACAACTCTTAATGACTTTAACCTTGTGGCTATGAAGTAC 10500

MT152900.1 ------------------------------------------------------------ 322

NC_045512.2:266-21555 AATTATGAACCTCTAACACAAGACCATGTTGACATACTAGGACCTCTTTCTGCTCAAACT 10560

MT152900.1 ------------------------------------------------------------ 322

NC_045512.2:266-21555 GGAATTGCCGTTTTAGATATGTGTGCTTCATTAAAAGAATTACTGCAAAATGGTATGAAT 10620

MT152900.1 ------------------------------------------------------------ 322

NC_045512.2:266-21555 GGACGTACCATATTGGGTAGTGCTTTATTAGAAGATGAATTTACACCTTTTGATGTTGTT 10680

MT152900.1 ------------------------------------------------------------ 322

NC_045512.2:266-21555 AGACAATGCTCAGGTGTTACTTTCCAAAGTGCAGTGAAAAGAACAATCAAGGGTACACAC 10740

MT152900.1 ------------------------------------------------------------ 322

NC_045512.2:266-21555 CACTGGTTGTTACTCACAATTTTGACTTCACTTTTAGTTTTAGTCCAGAGTACTCAATGG 10800

MT152900.1 ------------------------------------------------------------ 322

NC_045512.2:266-21555 TCTTTGTTCTTTTTTTTGTATGAAAATGCCTTTTTACCTTTTGCTATGGGTATTATTGCT 10860

MT152900.1 ------------------------------------------------------------ 322

NC_045512.2:266-21555 ATGTCTGCTTTTGCAATGATGTTTGTCAAACATAAGCATGCATTTCTCTGTTTGTTTTTG 10920

MT152900.1 ------------------------------------------------------------ 322

NC_045512.2:266-21555 TTACCTTCTCTTGCCACTGTAGCTTATTTTAATATGGTCTATATGCCTGCTAGTTGGGTG 10980

MT152900.1 ------------------------------------------------------------ 322

NC_045512.2:266-21555 ATGCGTATTATGACATGGTTGGATATGGTTGATACTAGTTTGTCTGGTTTTAAGCTAAAA 11040

MT152900.1 ------------------------------------------------------------ 322

NC_045512.2:266-21555 GACTGTGTTATGTATGCATCAGCTGTAGTGTTACTAATCCTTATGACAGCAAGAACTGTG 11100

MT152900.1 ------------------------------------------------------------ 322

NC_045512.2:266-21555 TATGATGATGGTGCTAGGAGAGTGTGGACACTTATGAATGTCTTGACACTCGTTTATAAA 11160

MT152900.1 ------------------------------------------------------------ 322

NC_045512.2:266-21555 GTTTATTATGGTAATGCTTTAGATCAAGCCATTTCCATGTGGGCTCTTATAATCTCTGTT 11220

MT152900.1 ------------------------------------------------------------ 322

NC_045512.2:266-21555 ACTTCTAACTACTCAGGTGTAGTTACAACTGTCATGTTTTTGGCCAGAGGTATTGTTTTT 11280

MT152900.1 ------------------------------------------------------------ 322

NC_045512.2:266-21555 ATGTGTGTTGAGTATTGCCCTATTTTCTTCATAACTGGTAATACACTTCAGTGTATAATG 11340

MT152900.1 ------------------------------------------------------------ 322

NC_045512.2:266-21555 CTAGTTTATTGTTTCTTAGGCTATTTTTGTACTTGTTACTTTGGCCTCTTTTGTTTACTC 11400

MT152900.1 ------------------------------------------------------------ 322

NC_045512.2:266-21555 AACCGCTACTTTAGACTGACTCTTGGTGTTTATGATTACTTAGTTTCTACACAGGAGTTT 11460

MT152900.1 ------------------------------------------------------------ 322

NC_045512.2:266-21555 AGATATATGAATTCACAGGGACTACTCCCACCCAAGAATAGCATAGATGCCTTCAAACTC 11520

MT152900.1 ------------------------------------------------------------ 322

NC_045512.2:266-21555 AACATTAAATTGTTGGGTGTTGGTGGCAAACCTTGTATCAAAGTAGCCACTGTACAGTCT 11580

MT152900.1 ------------------------------------------------------------ 322

NC_045512.2:266-21555 AAAATGTCAGATGTAAAGTGCACATCAGTAGTCTTACTCTCAGTTTTGCAACAACTCAGA 11640

MT152900.1 ------------------------------------------------------------ 322

NC_045512.2:266-21555 GTAGAATCATCATCTAAATTGTGGGCTCAATGTGTCCAGTTACACAATGACATTCTCTTA 11700

MT152900.1 ------------------------------------------------------------ 322

NC_045512.2:266-21555 GCTAAAGATACTACTGAAGCCTTTGAAAAAATGGTTTCACTACTTTCTGTTTTGCTTTCC 11760

MT152900.1 ------------------------------------------------------------ 322

NC_045512.2:266-21555 ATGCAGGGTGCTGTAGACATAAACAAGCTTTGTGAAGAAATGCTGGACAACAGGGCAACC 11820

MT152900.1 ------------------------------------------------------------ 322

NC_045512.2:266-21555 TTACAAGCTATAGCCTCAGAGTTTAGTTCCCTTCCATCATATGCAGCTTTTGCTACTGCT 11880

MT152900.1 ------------------------------------------------------------ 322

NC_045512.2:266-21555 CAAGAAGCTTATGAGCAGGCTGTTGCTAATGGTGATTCTGAAGTTGTTCTTAAAAAGTTG 11940

MT152900.1 ------------------------------------------------------------ 322

NC_045512.2:266-21555 AAGAAGTCTTTGAATGTGGCTAAATCTGAATTTGACCGTGATGCAGCCATGCAACGTAAG 12000

MT152900.1 ------------------------------------------------------------ 322

NC_045512.2:266-21555 TTGGAAAAGATGGCTGATCAAGCTATGACCCAAATGTATAAACAGGCTAGATCTGAGGAC 12060

MT152900.1 ------------------------------------------------------------ 322

NC_045512.2:266-21555 AAGAGGGCAAAAGTTACTAGTGCTATGCAGACAATGCTTTTCACTATGCTTAGAAAGTTG 12120

MT152900.1 ------------------------------------------------------------ 322

NC_045512.2:266-21555 GATAATGATGCACTCAACAACATTATCAACAATGCAAGAGATGGTTGTGTTCCCTTGAAC 12180

MT152900.1 ------------------------------------------------------------ 322

NC_045512.2:266-21555 ATAATACCTCTTACAACAGCAGCCAAACTAATGGTTGTCATACCAGACTATAACACATAT 12240

MT152900.1 ------------------------------------------------------------ 322

NC_045512.2:266-21555 AAAAATACGTGTGATGGTACAACATTTACTTATGCATCAGCATTGTGGGAAATCCAACAG 12300

MT152900.1 ------------------------------------------------------------ 322

NC_045512.2:266-21555 GTTGTAGATGCAGATAGTAAAATTGTTCAACTTAGTGAAATTAGTATGGACAATTCACCT 12360

MT152900.1 ------------------------------------------------------------ 322

NC_045512.2:266-21555 AATTTAGCATGGCCTCTTATTGTAACAGCTTTAAGGGCCAATTCTGCTGTCAAATTACAG 12420

MT152900.1 ------------------------------------------------------------ 322

NC_045512.2:266-21555 AATAATGAGCTTAGTCCTGTTGCACTACGACAGATGTCTTGTGCTGCCGGTACTACACAA 12480

MT152900.1 ------------------------------------------------------------ 322

NC_045512.2:266-21555 ACTGCTTGCACTGATGACAATGCGTTAGCTTACTACAACACAACAAAGGGAGGTAGGTTT 12540

MT152900.1 ------------------------------------------------------------ 322

NC_045512.2:266-21555 GTACTTGCACTGTTATCCGATTTACAGGATTTGAAATGGGCTAGATTCCCTAAGAGTGAT 12600

MT152900.1 ------------------------------------------------------------ 322

NC_045512.2:266-21555 GGAACTGGTACTATCTATACAGAACTGGAACCACCTTGTAGGTTTGTTACAGACACACCT 12660

MT152900.1 ------------------------------------------------------------ 322

NC_045512.2:266-21555 AAAGGTCCTAAAGTGAAGTATTTATACTTTATTAAAGGATTAAACAACCTAAATAGAGGT 12720

MT152900.1 ------------------------------------------------------------ 322

NC_045512.2:266-21555 ATGGTACTTGGTAGTTTAGCTGCCACAGTACGTCTACAAGCTGGTAATGCAACAGAAGTG 12780

MT152900.1 ------------------------------------------------------------ 322

NC_045512.2:266-21555 CCTGCCAATTCAACTGTATTATCTTTCTGTGCTTTTGCTGTAGATGCTGCTAAAGCTTAC 12840

MT152900.1 ------------------------------------------------------------ 322

NC_045512.2:266-21555 AAAGATTATCTAGCTAGTGGGGGACAACCAATCACTAATTGTGTTAAGATGTTGTGTACA 12900

MT152900.1 ------------------------------------------------------------ 322

NC_045512.2:266-21555 CACACTGGTACTGGTCAGGCAATAACAGTTACACCGGAAGCCAATATGGATCAAGAATCC 12960

MT152900.1 ------------------------------------------------------------ 322

NC_045512.2:266-21555 TTTGGTGGTGCATCGTGTTGTCTGTACTGCCGTTGCCACATAGATCATCCAAATCCTAAA 13020

MT152900.1 ------------------------------------------------------------ 322

NC_045512.2:266-21555 GGATTTTGTGACTTAAAAGGTAAGTATGTACAAATACCTACAACTTGTGCTAATGACCCT 13080

MT152900.1 ------------------------------------------------------------ 322

NC_045512.2:266-21555 GTGGGTTTTACACTTAAAAACACAGTCTGTACCGTCTGCGGTATGTGGAAAGGTTATGGC 13140

MT152900.1 ------------------------------------------------------------ 322

NC_045512.2:266-21555 TGTAGTTGTGATCAACTCCGCGAACCCATGCTTCAGTCAGCTGATGCACAATCGTTTTTA 13200

MT152900.1 ------------------------------------------------------------ 322

NC_045512.2:266-21555 AACGGGTTTGCGGTGTAAGTGCAGCCCGTCTTACACCGTGCGGCACAGGCACTAGTACTG 13260

MT152900.1 ------------------------------------------------------------ 322

NC_045512.2:266-21555 ATGTCGTATACAGGGCTTTTGACATCTACAATGATAAAGTAGCTGGTTTTGCTAAATTCC 13320

MT152900.1 ------------------------------------------------------------ 322

NC_045512.2:266-21555 TAAAAACTAATTGTTGTCGCTTCCAAGAAAAGGACGAAGATGACAATTTAATTGATTCTT 13380

MT152900.1 ------------------------------------------------------------ 322

NC_045512.2:266-21555 ACTTTGTAGTTAAGAGACACACTTTCTCTAACTACCAACATGAAGAAACAATTTATAATT 13440

MT152900.1 ------------------------------------------------------------ 322

NC_045512.2:266-21555 TACTTAAGGATTGTCCAGCTGTTGCTAAACATGACTTCTTTAAGTTTAGAATAGACGGTG 13500

MT152900.1 ------------------------------------------------------------ 322

NC_045512.2:266-21555 ACATGGTACCACATATATCACGTCAACGTCTTACTAAATACACAATGGCAGACCTCGTCT 13560

MT152900.1 ------------------------------------------------------------ 322

NC_045512.2:266-21555 ATGCTTTAAGGCATTTTGATGAAGGTAATTGTGACACATTAAAAGAAATACTTGTCACAT 13620

MT152900.1 ------------------------------------------------------------ 322

NC_045512.2:266-21555 ACAATTGTTGTGATGATGATTATTTCAATAAAAAGGACTGGTATGATTTTGTAGAAAACC 13680

MT152900.1 ------------------------------------------------------------ 322

NC_045512.2:266-21555 CAGATATATTACGCGTATACGCCAACTTAGGTGAACGTGTACGCCAAGCTTTGTTAAAAA 13740

MT152900.1 ------------------------------------------------------------ 322

NC_045512.2:266-21555 CAGTACAATTCTGTGATGCCATGCGAAATGCTGGTATTGTTGGTGTACTGACATTAGATA 13800

MT152900.1 ------------------------------------------------------------ 322

NC_045512.2:266-21555 ATCAAGATCTCAATGGTAACTGGTATGATTTCGGTGATTTCATACAAACCACGCCAGGTA 13860

MT152900.1 ------------------------------------------------------------ 322

NC_045512.2:266-21555 GTGGAGTTCCTGTTGTAGATTCTTATTATTCATTGTTAATGCCTATATTAACCTTGACCA 13920

MT152900.1 ------------------------------------------------------------ 322

NC_045512.2:266-21555 GGGCTTTAACTGCAGAGTCACATGTTGACACTGACTTAACAAAGCCTTACATTAAGTGGG 13980

MT152900.1 ------------------------------------------------------------ 322

NC_045512.2:266-21555 ATTTGTTAAAATATGACTTCACGGAAGAGAGGTTAAAACTCTTTGACCGTTATTTTAAAT 14040

MT152900.1 ------------------------------------------------------------ 322

NC_045512.2:266-21555 ATTGGGATCAGACATACCACCCAAATTGTGTTAACTGTTTGGATGACAGATGCATTCTGC 14100

MT152900.1 ------------------------------------------------------------ 322

NC_045512.2:266-21555 ATTGTGCAAACTTTAATGTTTTATTCTCTACAGTGTTCCCACCTACAAGTTTTGGACCAC 14160

MT152900.1 ------------------------------------------------------------ 322

NC_045512.2:266-21555 TAGTGAGAAAAATATTTGTTGATGGTGTTCCATTTGTAGTTTCAACTGGATACCACTTCA 14220

MT152900.1 ------------------------------------------------------------ 322

NC_045512.2:266-21555 GAGAGCTAGGTGTTGTACATAATCAGGATGTAAACTTACATAGCTCTAGACTTAGTTTTA 14280

MT152900.1 ------------------------------------------------------------ 322

NC_045512.2:266-21555 AGGAATTACTTGTGTATGCTGCTGACCCTGCTATGCACGCTGCTTCTGGTAATCTATTAC 14340

MT152900.1 ------------------------------------------------------------ 322

NC_045512.2:266-21555 TAGATAAACGCACTACGTGCTTTTCAGTAGCTGCACTTACTAACAATGTTGCTTTTCAAA 14400

MT152900.1 ------------------------------------------------------------ 322

NC_045512.2:266-21555 CTGTCAAACCCGGTAATTTTAACAAAGACTTCTATGACTTTGCTGTGTCTAAGGGTTTCT 14460

MT152900.1 ------------------------------------------------------------ 322

NC_045512.2:266-21555 TTAAGGAAGGAAGTTCTGTTGAATTAAAACACTTCTTCTTTGCTCAGGATGGTAATGCTG 14520

MT152900.1 ------------------------------------------------------------ 322

NC_045512.2:266-21555 CTATCAGCGATTATGACTACTATCGTTATAATCTACCAACAATGTGTGATATCAGACAAC 14580

MT152900.1 ------------------------------------------------------------ 322

NC_045512.2:266-21555 TACTATTTGTAGTTGAAGTTGTTGATAAGTACTTTGATTGTTACGATGGTGGCTGTATTA 14640

MT152900.1 ------------------------------------------------------------ 322

NC_045512.2:266-21555 ATGCTAACCAAGTCATCGTCAACAACCTAGACAAATCAGCTGGTTTTCCATTTAATAAAT 14700

MT152900.1 ------------------------------------------------------------ 322

NC_045512.2:266-21555 GGGGTAAGGCTAGACTTTATTATGATTCAATGAGTTATGAGGATCAAGATGCACTTTTCG 14760

MT152900.1 ------------------------------------------------------------ 322

NC_045512.2:266-21555 CATATACAAAACGTAATGTCATCCCTACTATAACTCAAATGAATCTTAAGTATGCCATTA 14820

MT152900.1 ------------------------------------------------------------ 322

NC_045512.2:266-21555 GTGCAAAGAATAGAGCTCGCACCGTAGCTGGTGTCTCTATCTGTAGTACTATGACCAATA 14880

MT152900.1 ------------------------------------------------------------ 322

NC_045512.2:266-21555 GACAGTTTCATCAAAAATTATTGAAATCAATAGCCGCCACTAGAGGAGCTACTGTAGTAA 14940

MT152900.1 ------------------------------------------------------------ 322

NC_045512.2:266-21555 TTGGAACAAGCAAATTCTATGGTGGTTGGCACAACATGTTAAAAACTGTTTATAGTGATG 15000

MT152900.1 ------------------------------------------------------------ 322

NC_045512.2:266-21555 TAGAAAACCCTCACCTTATGGGTTGGGATTATCCTAAATGTGATAGAGCCATGCCTAACA 15060

MT152900.1 ------------------------------------------------------------ 322

NC_045512.2:266-21555 TGCTTAGAATTATGGCCTCACTTGTTCTTGCTCGCAAACATACAACGTGTTGTAGCTTGT 15120

MT152900.1 ------------------------------------------------------------ 322

NC_045512.2:266-21555 CACACCGTTTCTATAGATTAGCTAATGAGTGTGCTCAAGTATTGAGTGAAATGGTCATGT 15180

MT152900.1 ------------------------------------------------------------ 322

NC_045512.2:266-21555 GTGGCGGTTCACTATATGTTAAACCAGGTGGAACCTCATCAGGAGATGCCACAACTGCTT 15240

MT152900.1 ------------------------------------------------------------ 322

NC_045512.2:266-21555 ATGCTAATAGTGTTTTTAACATTTGTCAAGCTGTCACGGCCAATGTTAATGCACTTTTAT 15300

MT152900.1 ------------------------------------------------------------ 322

NC_045512.2:266-21555 CTACTGATGGTAACAAAATTGCCGATAAGTATGTCCGCAATTTACAACACAGACTTTATG 15360

MT152900.1 ------------------------------------------------------------ 322

NC_045512.2:266-21555 AGTGTCTCTATAGAAATAGAGATGTTGACACAGACTTTGTGAATGAGTTTTACGCATATT 15420

MT152900.1 ------------------------------------------------------------ 322

NC_045512.2:266-21555 TGCGTAAACATTTCTCAATGATGATACTCTCTGACGATGCTGTTGTGTGTTTCAATAGCA 15480

MT152900.1 ------------------------------------------------------------ 322

NC_045512.2:266-21555 CTTATGCATCTCAAGGTCTAGTGGCTAGCATAAAGAACTTTAAGTCAGTTCTTTATTATC 15540

MT152900.1 ------------------------------------------------------------ 322

NC_045512.2:266-21555 AAAACAATGTTTTTATGTCTGAAGCAAAATGTTGGACTGAGACTGACCTTACTAAAGGAC 15600

MT152900.1 ------------------------------------------------------------ 322

NC_045512.2:266-21555 CTCATGAATTTTGCTCTCAACATACAATGCTAGTTAAACAGGGTGATGATTATGTGTACC 15660

MT152900.1 ------------------------------------------------------------ 322

NC_045512.2:266-21555 TTCCTTACCCAGATCCATCAAGAATCCTAGGGGCCGGCTGTTTTGTAGATGATATCGTAA 15720

MT152900.1 ------------------------------------------------------------ 322

NC_045512.2:266-21555 AAACAGATGGTACACTTATGATTGAACGGTTCGTGTCTTTAGCTATAGATGCTTACCCAC 15780

MT152900.1 ------------------------------------------------------------ 322

NC_045512.2:266-21555 TTACTAAACATCCTAATCAGGAGTATGCTGATGTCTTTCATTTGTACTTACAATACATAA 15840

MT152900.1 ------------------------------------------------------------ 322

NC_045512.2:266-21555 GAAAGCTACATGATGAGTTAACAGGACACATGTTAGACATGTATTCTGTTATGCTTACTA 15900

MT152900.1 ------------------------------------------------------------ 322

NC_045512.2:266-21555 ATGATAACACTTCAAGGTATTGGGAACCTGAGTTTTATGAGGCTATGTACACACCGCATA 15960

MT152900.1 ------------------------------------------------------------ 322

NC_045512.2:266-21555 CAGTCTTACAGGCTGTTGGGGCTTGTGTTCTTTGCAATTCACAGACTTCATTAAGATGTG 16020

MT152900.1 ------------------------------------------------------------ 322

NC_045512.2:266-21555 GTGCTTGCATACGTAGACCATTCTTATGTTGTAAATGCTGTTACGACCATGTCATATCAA 16080

MT152900.1 ------------------------------------------------------------ 322

NC_045512.2:266-21555 CATCACATAAATTAGTCTTGTCTGTTAATCCGTATGTTTGCAATGCTCCAGGTTGTGATG 16140

MT152900.1 ------------------------------------------------------------ 322

NC_045512.2:266-21555 TCACAGATGTGACTCAACTTTACTTAGGAGGTATGAGCTATTATTGTAAATCACATAAAC 16200

MT152900.1 ------------------------------------------------------------ 322

NC_045512.2:266-21555 CACCCATTAGTTTTCCATTGTGTGCTAATGGACAAGTTTTTGGTTTATATAAAAATACAT 16260

MT152900.1 ------------------------------------------------------------ 322

NC_045512.2:266-21555 GTGTTGGTAGCGATAATGTTACTGACTTTAATGCAATTGCAACATGTGACTGGACAAATG 16320

MT152900.1 ------------------------------------------------------------ 322

NC_045512.2:266-21555 CTGGTGATTACATTTTAGCTAACACCTGTACTGAAAGACTCAAGCTTTTTGCAGCAGAAA 16380

MT152900.1 ------------------------------------------------------------ 322

NC_045512.2:266-21555 CGCTCAAAGCTACTGAGGAGACATTTAAACTGTCTTATGGTATTGCTACTGTACGTGAAG 16440

MT152900.1 ------------------------------------------------------------ 322

NC_045512.2:266-21555 TGCTGTCTGACAGAGAATTACATCTTTCATGGGAAGTTGGTAAACCTAGACCACCACTTA 16500

MT152900.1 ------------------------------------------------------------ 322

NC_045512.2:266-21555 ACCGAAATTATGTCTTTACTGGTTATCGTGTAACTAAAAACAGTAAAGTACAAATAGGAG 16560

MT152900.1 ------------------------------------------------------------ 322

NC_045512.2:266-21555 AGTACACCTTTGAAAAAGGTGACTATGGTGATGCTGTTGTTTACCGAGGTACAACAACTT 16620

MT152900.1 ------------------------------------------------------------ 322

NC_045512.2:266-21555 ACAAATTAAATGTTGGTGATTATTTTGTGCTGACATCACATACAGTAATGCCATTAAGTG 16680

MT152900.1 ------------------------------------------------------------ 322

NC_045512.2:266-21555 CACCTACACTAGTGCCACAAGAGCACTATGTTAGAATTACTGGCTTATACCCAACACTCA 16740

MT152900.1 ------------------------------------------------------------ 322

NC_045512.2:266-21555 ATATCTCAGATGAGTTTTCTAGCAATGTTGCAAATTATCAAAAGGTTGGTATGCAAAAGT 16800

MT152900.1 ------------------------------------------------------------ 322

NC_045512.2:266-21555 ATTCTACACTCCAGGGACCACCTGGTACTGGTAAGAGTCATTTTGCTATTGGCCTAGCTC 16860

MT152900.1 ------------------------------------------------------------ 322

NC_045512.2:266-21555 TCTACTACCCTTCTGCTCGCATAGTGTATACAGCTTGCTCTCATGCCGCTGTTGATGCAC 16920

MT152900.1 ------------------------------------------------------------ 322

NC_045512.2:266-21555 TATGTGAGAAGGCATTAAAATATTTGCCTATAGATAAATGTAGTAGAATTATACCTGCAC 16980

MT152900.1 ------------------------------------------------------------ 322

NC_045512.2:266-21555 GTGCTCGTGTAGAGTGTTTTGATAAATTCAAAGTGAATTCAACATTAGAACAGTATGTCT 17040

MT152900.1 ------------------------------------------------------------ 322

NC_045512.2:266-21555 TTTGTACTGTAAATGCATTGCCTGAGACGACAGCAGATATAGTTGTCTTTGATGAAATTT 17100

MT152900.1 ------------------------------------------------------------ 322

NC_045512.2:266-21555 CAATGGCCACAAATTATGATTTGAGTGTTGTCAATGCCAGATTACGTGCTAAGCACTATG 17160

MT152900.1 ------------------------------------------------------------ 322

NC_045512.2:266-21555 TGTACATTGGCGACCCTGCTCAATTACCTGCACCACGCACATTGCTAACTAAGGGCACAC 17220

MT152900.1 ------------------------------------------------------------ 322

NC_045512.2:266-21555 TAGAACCAGAATATTTCAATTCAGTGTGTAGACTTATGAAAACTATAGGTCCAGACATGT 17280

MT152900.1 ------------------------------------------------------------ 322

NC_045512.2:266-21555 TCCTCGGAACTTGTCGGCGTTGTCCTGCTGAAATTGTTGACACTGTGAGTGCTTTGGTTT 17340

MT152900.1 ------------------------------------------------------------ 322

NC_045512.2:266-21555 ATGATAATAAGCTTAAAGCACATAAAGACAAATCAGCTCAATGCTTTAAAATGTTTTATA 17400

MT152900.1 ------------------------------------------------------------ 322

NC_045512.2:266-21555 AGGGTGTTATCACGCATGATGTTTCATCTGCAATTAACAGGCCACAAATAGGCGTGGTAA 17460

MT152900.1 ------------------------------------------------------------ 322

NC_045512.2:266-21555 GAGAATTCCTTACACGTAACCCTGCTTGGAGAAAAGCTGTCTTTATTTCACCTTATAATT 17520

MT152900.1 ------------------------------------------------------------ 322

NC_045512.2:266-21555 CACAGAATGCTGTAGCCTCAAAGATTTTGGGACTACCAACTCAAACTGTTGATTCATCAC 17580

MT152900.1 ------------------------------------------------------------ 322

NC_045512.2:266-21555 AGGGCTCAGAATATGACTATGTCATATTCACTCAAACCACTGAAACAGCTCACTCTTGTA 17640

MT152900.1 ------------------------------------------------------------ 322

NC_045512.2:266-21555 ATGTAAACAGATTTAATGTTGCTATTACCAGAGCAAAAGTAGGCATACTTTGCATAATGT 17700

MT152900.1 ------------------------------------------------------------ 322

NC_045512.2:266-21555 CTGATAGAGACCTTTATGACAAGTTGCAATTTACAAGTCTTGAAATTCCACGTAGGAATG 17760

MT152900.1 ------------------------------------------------------------ 322

NC_045512.2:266-21555 TGGCAACTTTACAAGCTGAAAATGTAACAGGACTCTTTAAAGATTGTAGTAAGGTAATCA 17820

MT152900.1 ------------------------------------------------------------ 322

NC_045512.2:266-21555 CTGGGTTACATCCTACACAGGCACCTACACACCTCAGTGTTGACACTAAATTCAAAACTG 17880

MT152900.1 ------------------------------------------------------------ 322

NC_045512.2:266-21555 AAGGTTTATGTGTTGACATACCTGGCATACCTAAGGACATGACCTATAGAAGACTCATCT 17940

MT152900.1 ------------------------------------------------------------ 322

NC_045512.2:266-21555 CTATGATGGGTTTTAAAATGAATTATCAAGTTAATGGTTACCCTAACATGTTTATCACCC 18000

MT152900.1 ------------------------------------------------------------ 322

NC_045512.2:266-21555 GCGAAGAAGCTATAAGACATGTACGTGCATGGATTGGCTTCGATGTCGAGGGGTGTCATG 18060

MT152900.1 ------------------------------------------------------------ 322

NC_045512.2:266-21555 CTACTAGAGAAGCTGTTGGTACCAATTTACCTTTACAGCTAGGTTTTTCTACAGGTGTTA 18120

MT152900.1 ------------------------------------------------------------ 322

NC_045512.2:266-21555 ACCTAGTTGCTGTACCTACAGGTTATGTTGATACACCTAATAATACAGATTTTTCCAGAG 18180

MT152900.1 ------------------------------------------------------------ 322

NC_045512.2:266-21555 TTAGTGCTAAACCACCGCCTGGAGATCAATTTAAACACCTCATACCACTTATGTACAAAG 18240

MT152900.1 ------------------------------------------------------------ 322

NC_045512.2:266-21555 GACTTCCTTGGAATGTAGTGCGTATAAAGATTGTACAAATGTTAAGTGACACACTTAAAA 18300

MT152900.1 ------------------------------------------------------------ 322

NC_045512.2:266-21555 ATCTCTCTGACAGAGTCGTATTTGTCTTATGGGCACATGGCTTTGAGTTGACATCTATGA 18360

MT152900.1 ------------------------------------------------------------ 322

NC_045512.2:266-21555 AGTATTTTGTGAAAATAGGACCTGAGCGCACCTGTTGTCTATGTGATAGACGTGCCACAT 18420

MT152900.1 ------------------------------------------------------------ 322

NC_045512.2:266-21555 GCTTTTCCACTGCTTCAGACACTTATGCCTGTTGGCATCATTCTATTGGATTTGATTACG 18480

MT152900.1 ------------------------------------------------------------ 322

NC_045512.2:266-21555 TCTATAATCCGTTTATGATTGATGTTCAACAATGGGGTTTTACAGGTAACCTACAAAGCA 18540

MT152900.1 ------------------------------------------------------------ 322

NC_045512.2:266-21555 ACCATGATCTGTATTGTCAAGTCCATGGTAATGCACATGTAGCTAGTTGTGATGCAATCA 18600

MT152900.1 ------------------------------------------------------------ 322

NC_045512.2:266-21555 TGACTAGGTGTCTAGCTGTCCACGAGTGCTTTGTTAAGCGTGTTGACTGGACTATTGAAT 18660

MT152900.1 ------------------------------------------------------------ 322

NC_045512.2:266-21555 ATCCTATAATTGGTGATGAACTGAAGATTAATGCGGCTTGTAGAAAGGTTCAACACATGG 18720

MT152900.1 ------------------------------------------------------------ 322

NC_045512.2:266-21555 TTGTTAAAGCTGCATTATTAGCAGACAAATTCCCAGTTCTTCACGACATTGGTAACCCTA 18780

MT152900.1 ------------------------------------------------------------ 322

NC_045512.2:266-21555 AAGCTATTAAGTGTGTACCTCAAGCTGATGTAGAATGGAAGTTCTATGATGCACAGCCTT 18840

MT152900.1 ------------------------------------------------------------ 322

NC_045512.2:266-21555 GTAGTGACAAAGCTTATAAAATAGAAGAATTATTCTATTCTTATGCCACACATTCTGACA 18900

MT152900.1 ------------------------------------------------------------ 322

NC_045512.2:266-21555 AATTCACAGATGGTGTATGCCTATTTTGGAATTGCAATGTCGATAGATATCCTGCTAATT 18960

MT152900.1 ------------------------------------------------------------ 322

NC_045512.2:266-21555 CCATTGTTTGTAGATTTGACACTAGAGTGCTATCTAACCTTAACTTGCCTGGTTGTGATG 19020

MT152900.1 ------------------------------------------------------------ 322

NC_045512.2:266-21555 GTGGCAGTTTGTATGTAAATAAACATGCATTCCACACACCAGCTTTTGATAAAAGTGCTT 19080

MT152900.1 ------------------------------------------------------------ 322

NC_045512.2:266-21555 TTGTTAATTTAAAACAATTACCATTTTTCTATTACTCTGACAGTCCATGTGAGTCTCATG 19140

MT152900.1 ------------------------------------------------------------ 322

NC_045512.2:266-21555 GAAAACAAGTAGTGTCAGATATAGATTATGTACCACTAAAGTCTGCTACGTGTATAACAC 19200

MT152900.1 ------------------------------------------------------------ 322

NC_045512.2:266-21555 GTTGCAATTTAGGTGGTGCTGTCTGTAGACATCATGCTAATGAGTACAGATTGTATCTCG 19260

MT152900.1 ------------------------------------------------------------ 322

NC_045512.2:266-21555 ATGCTTATAACATGATGATCTCAGCTGGCTTTAGCTTGTGGGTTTACAAACAATTTGATA 19320

MT152900.1 ------------------------------------------------------------ 322

NC_045512.2:266-21555 CTTATAACCTCTGGAACACTTTTACAAGACTTCAGAGTTTAGAAAATGTGGCTTTTAATG 19380

MT152900.1 ------------------------------------------------------------ 322

NC_045512.2:266-21555 TTGTAAATAAGGGACACTTTGATGGACAACAGGGTGAAGTACCAGTTTCTATCATTAATA 19440

MT152900.1 ------------------------------------------------------------ 322

NC_045512.2:266-21555 ACACTGTTTACACAAAAGTTGATGGTGTTGATGTAGAATTGTTTGAAAATAAAACAACAT 19500

MT152900.1 ------------------------------------------------------------ 322

NC_045512.2:266-21555 TACCTGTTAATGTAGCATTTGAGCTTTGGGCTAAGCGCAACATTAAACCAGTACCAGAGG 19560

MT152900.1 ------------------------------------------------------------ 322

NC_045512.2:266-21555 TGAAAATACTCAATAATTTGGGTGTGGACATTGCTGCTAATACTGTGATCTGGGACTACA 19620

MT152900.1 ------------------------------------------------------------ 322

NC_045512.2:266-21555 AAAGAGATGCTCCAGCACATATATCTACTATTGGTGTTTGTTCTATGACTGACATAGCCA 19680

MT152900.1 ------------------------------------------------------------ 322

NC_045512.2:266-21555 AGAAACCAACTGAAACGATTTGTGCACCACTCACTGTCTTTTTTGATGGTAGAGTTGATG 19740

MT152900.1 ------------------------------------------------------------ 322

NC_045512.2:266-21555 GTCAAGTAGACTTATTTAGAAATGCCCGTAATGGTGTTCTTATTACAGAAGGTAGTGTTA 19800

MT152900.1 ------------------------------------------------------------ 322

NC_045512.2:266-21555 AAGGTTTACAACCATCTGTAGGTCCCAAACAAGCTAGTCTTAATGGAGTCACATTAATTG 19860

MT152900.1 ------------------------------------------------------------ 322

NC_045512.2:266-21555 GAGAAGCCGTAAAAACACAGTTCAATTATTATAAGAAAGTTGATGGTGTTGTCCAACAAT 19920

MT152900.1 ------------------------------------------------------------ 322

NC_045512.2:266-21555 TACCTGAAACTTACTTTACTCAGAGTAGAAATTTACAAGAATTTAAACCCAGGAGTCAAA 19980

MT152900.1 ------------------------------------------------------------ 322

NC_045512.2:266-21555 TGGAAATTGATTTCTTAGAATTAGCTATGGATGAATTCATTGAACGGTATAAATTAGAAG 20040

MT152900.1 ------------------------------------------------------------ 322

NC_045512.2:266-21555 GCTATGCCTTCGAACATATCGTTTATGGAGATTTTAGTCATAGTCAGTTAGGTGGTTTAC 20100

MT152900.1 ------------------------------------------------------------ 322

NC_045512.2:266-21555 ATCTACTGATTGGACTAGCTAAACGTTTTAAGGAATCACCTTTTGAATTAGAAGATTTTA 20160

MT152900.1 ------------------------------------------------------------ 322

NC_045512.2:266-21555 TTCCTATGGACAGTACAGTTAAAAACTATTTCATAACAGATGCGCAAACAGGTTCATCTA 20220

MT152900.1 ------------------------------------------------------------ 322

NC_045512.2:266-21555 AGTGTGTGTGTTCTGTTATTGATTTATTACTTGATGATTTTGTTGAAATAATAAAATCCC 20280

MT152900.1 ------------------------------------------------------------ 322

NC_045512.2:266-21555 AAGATTTATCTGTAGTTTCTAAGGTTGTCAAAGTGACTATTGACTATACAGAAATTTCAT 20340

MT152900.1 ------------------------------------------------------------ 322

NC_045512.2:266-21555 TTATGCTTTGGTGTAAAGATGGCCATGTAGAAACATTTTACCCAAAATTACAATCTAGTC 20400

MT152900.1 ------------------------------------------------------------ 322

NC_045512.2:266-21555 AAGCGTGGCAACCGGGTGTTGCTATGCCTAATCTTTACAAAATGCAAAGAATGCTATTAG 20460

MT152900.1 ------------------------------------------------------------ 322

NC_045512.2:266-21555 AAAAGTGTGACCTTCAAAATTATGGTGATAGTGCAACATTACCTAAAGGCATAATGATGA 20520

MT152900.1 ------------------------------------------------------------ 322

NC_045512.2:266-21555 ATGTCGCAAAATATACTCAACTGTGTCAATATTTAAACACATTAACATTAGCTGTACCCT 20580

MT152900.1 ------------------------------------------------------------ 322

NC_045512.2:266-21555 ATAATATGAGAGTTATACATTTTGGTGCTGGTTCTGATAAAGGAGTTGCACCAGGTACAG 20640

MT152900.1 ------------------------------------------------------------ 322

NC_045512.2:266-21555 CTGTTTTAAGACAGTGGTTGCCTACGGGTACGCTGCTTGTCGATTCAGATCTTAATGACT 20700

MT152900.1 ------------------------------------------------------------ 322

NC_045512.2:266-21555 TTGTCTCTGATGCAGATTCAACTTTGATTGGTGATTGTGCAACTGTACATACAGCTAATA 20760

MT152900.1 ------------------------------------------------------------ 322

NC_045512.2:266-21555 AATGGGATCTCATTATTAGTGATATGTACGACCCTAAGACTAAAAATGTTACAAAAGAAA 20820

MT152900.1 ------------------------------------------------------------ 322

NC_045512.2:266-21555 ATGACTCTAAAGAGGGTTTTTTCACTTACATTTGTGGGTTTATACAACAAAAGCTAGCTC 20880

MT152900.1 ------------------------------------------------------------ 322

NC_045512.2:266-21555 TTGGAGGTTCCGTGGCTATAAAGATAACAGAACATTCTTGGAATGCTGATCTTTATAAGC 20940

MT152900.1 ------------------------------------------------------------ 322

NC_045512.2:266-21555 TCATGGGACACTTCGCATGGTGGACAGCCTTTGTTACTAATGTGAATGCGTCATCATCTG 21000

MT152900.1 ------------------------------------------------------------ 322

NC_045512.2:266-21555 AAGCATTTTTAATTGGATGTAATTATCTTGGCAAACCACGCGAACAAATAGATGGTTATG 21060

MT152900.1 ------------------------------------------------------------ 322

NC_045512.2:266-21555 TCATGCATGCAAATTACATATTTTGGAGGAATACAAATCCAATTCAGTTGTCTTCCTATT 21120

MT152900.1 ------------------------------------------------------------ 322

NC_045512.2:266-21555 CTTTATTTGACATGAGTAAATTTCCCCTTAAATTAAGGGGTACTGCTGTTATGTCTTTAA 21180

MT152900.1 ------------------------------------------------------------ 322

NC_045512.2:266-21555 AAGAAGGTCAAATCAATGATATGATTTTATCTCTTCTTAGTAAAGGTAGACTTATAATTA 21240

MT152900.1 ------------------------------------------------------------ 322

NC_045512.2:266-21555 GAGAAAACAACAGAGTTGTTATTTCTAGTGATGTTCTTGTTAACAACTAA 21290

MT152900.1 -------------------------------------------------- 322

**B. MSA between the full ORF1ab protein sequence from Wuhan, China, and partial ORF1ab protein sequence from Iran**

CLUSTAL O(1.2.4) multiple sequence alignment

QIH55230.1 ------------------------------------------------------------ 0

YP_009724389.1 MESLVPGFNEKTHVQLSLPVLQVRDVLVRGFGDSVEEVLSEARQHLKDGTCGLVEVEKGV 60

QIH55230.1 -------------------PHGHVMVELVAELEGIQYGRSGETLGVLVPHVGEIPVAYRK 41

YP_009724389.1 LPQLEQPYVFIKRSDARTAPHGHVMVELVAELEGIQYGRSGETLGVLVPHVGEIPVAYRK 120

*****************************************

QIH55230.1 VLLRKNGNKGAGGHSYGADLKSFDLGDELGTDPYEDFQENWNTKHSSGVTRELMRELNGG 101

YP_009724389.1 VLLRKNGNKGAGGHSYGADLKSFDLGDELGTDPYEDFQENWNTKHSSGVTRELMRELNGG 180

************************************************************

QIH55230.1 AYTRYV------------------------------------------------------ 107

YP_009724389.1 AYTRYVDNNFCGPDGYPLECIKDLLARAGKASCTLSEQLDFIDTKRGVYCCREHEHEIAW 240

******

QIH55230.1 ------------------------------------------------------------ 107

YP_009724389.1 YTERSEKSYELQTPFEIKLAKKFDTFNGECPNFVFPLNSIIKTIQPRVEKKKLDGFMGRI 300

QIH55230.1 ------------------------------------------------------------ 107

YP_009724389.1 RSVYPVASPNECNQMCLSTLMKCDHCGETSWQTGDFVKATCEFCGTENLTKEGATTCGYL 360

QIH55230.1 ------------------------------------------------------------ 107

YP_009724389.1 PQNAVVKIYCPACHNSEVGPEHSLAEYHNESGLKTILRKGGRTIAFGGCVFSYVGCHNKC 420

QIH55230.1 ------------------------------------------------------------ 107

YP_009724389.1 AYWVPRASANIGCNHTGVVGEGSEGLNDNLLEILQKEKVNINIVGDFKLNEEIAIILASF 480

QIH55230.1 ------------------------------------------------------------ 107

YP_009724389.1 SASTSAFVETVKGLDYKAFKQIVESCGNFKVTKGKAKKGAWNIGEQKSILSPLYAFASEA 540

QIH55230.1 ------------------------------------------------------------ 107

YP_009724389.1 ARVVRSIFSRTLETAQNSVRVLQKAAITILDGISQYSLRLIDAMMFTSDLATNNLVVMAY 600

QIH55230.1 ------------------------------------------------------------ 107

YP_009724389.1 ITGGVVQLTSQWLTNIFGTVYEKLKPVLDWLEEKFKEGVEFLRDGWEIVKFISTCACEIV 660

QIH55230.1 ------------------------------------------------------------ 107

YP_009724389.1 GGQIVTCAKEIKESVQTFFKLVNKFLALCADSIIIGGAKLKALNLGETFVTHSKGLYRKC 720

QIH55230.1 ------------------------------------------------------------ 107

YP_009724389.1 VKSREETGLLMPLKAPKEIIFLEGETLPTEVLTEEVVLKTGDLQPLEQPTSEAVEAPLVG 780

QIH55230.1 ------------------------------------------------------------ 107

YP_009724389.1 TPVCINGLMLLEIKDTEKYCALAPNMMVTNNTFTLKGGAPTKVTFGDDTVIEVQGYKSVN 840

QIH55230.1 ------------------------------------------------------------ 107

YP_009724389.1 ITFELDERIDKVLNEKCSAYTVELGTEVNEFACVVADAVIKTLQPVSELLTPLGIDLDEW 900

QIH55230.1 ------------------------------------------------------------ 107

YP_009724389.1 SMATYYLFDESGEFKLASHMYCSFYPPDEDEEEGDCEEEEFEPSTQYEYGTEDDYQGKPL 960

QIH55230.1 ------------------------------------------------------------ 107

YP_009724389.1 EFGATSAALQPEEEQEEDWLDDDSQQTVGQQDGSEDNQTTTIQTIVEVQPQLEMELTPVV 1020

QIH55230.1 ------------------------------------------------------------ 107

YP_009724389.1 QTIEVNSFSGYLKLTDNVYIKNADIVEEAKKVKPTVVVNAANVYLKHGGGVAGALNKATN 1080

QIH55230.1 ------------------------------------------------------------ 107

YP_009724389.1 NAMQVESDDYIATNGPLKVGGSCVLSGHNLAKHCLHVVGPNVNKGEDIQLLKSAYENFNQ 1140

QIH55230.1 ------------------------------------------------------------ 107

YP_009724389.1 HEVLLAPLLSAGIFGADPIHSLRVCVDTVRTNVYLAVFDKNLYDKLVSSFLEMKSEKQVE 1200

QIH55230.1 ------------------------------------------------------------ 107

YP_009724389.1 QKIAEIPKEEVKPFITESKPSVEQRKQDDKKIKACVEEVTTTLEETKFLTENLLLYIDIN 1260

QIH55230.1 ------------------------------------------------------------ 107

YP_009724389.1 GNLHPDSATLVSDIDITFLKKDAPYIVGDVVQEGVLTAVVIPTKKAGGTTEMLAKALRKV 1320

QIH55230.1 ------------------------------------------------------------ 107

YP_009724389.1 PTDNYITTYPGQGLNGYTVEEAKTVLKKCKSAFYILPSIISNEKQEILGTVSWNLREMLA 1380

QIH55230.1 ------------------------------------------------------------ 107

YP_009724389.1 HAEETRKLMPVCVETKAIVSTIQRKYKGIKIQEGVVDYGARFYFYTSKTTVASLINTLND 1440

QIH55230.1 ------------------------------------------------------------ 107

YP_009724389.1 LNETLVTMPLGYVTHGLNLEEAARYMRSLKVPATVSVSSPDAVTAYNGYLTSSSKTPEEH 1500

QIH55230.1 ------------------------------------------------------------ 107

YP_009724389.1 FIETISLAGSYKDWSYSGQSTQLGIEFLKRGDKSVYYTSNPTTFHLDGEVITFDNLKTLL 1560

QIH55230.1 ------------------------------------------------------------ 107

YP_009724389.1 SLREVRTIKVFTTVDNINLHTQVVDMSMTYGQQFGPTYLDGADVTKIKPHNSHEGKTFYV 1620

QIH55230.1 ------------------------------------------------------------ 107

YP_009724389.1 LPNDDTLRVEAFEYYHTTDPSFLGRYMSALNHTKKWKYPQVNGLTSIKWADNNCYLATAL 1680

QIH55230.1 ------------------------------------------------------------ 107

YP_009724389.1 LTLQQIELKFNPPALQDAYYRARAGEAANFCALILAYCNKTVGELGDVRETMSYLFQHAN 1740

QIH55230.1 ------------------------------------------------------------ 107

YP_009724389.1 LDSCKRVLNVVCKTCGQQQTTLKGVEAVMYMGTLSYEQFKKGVQIPCTCGKQATKYLVQQ 1800

QIH55230.1 ------------------------------------------------------------ 107

YP_009724389.1 ESPFVMMSAPPAQYELKHGTFTCASEYTGNYQCGHYKHITSKETLYCIDGALLTKSSEYK 1860

QIH55230.1 ------------------------------------------------------------ 107

YP_009724389.1 GPITDVFYKENSYTTTIKPVTYKLDGVVCTEIDPKLDNYYKKDNSYFTEQPIDLVPNQPY 1920

QIH55230.1 ------------------------------------------------------------ 107

YP_009724389.1 PNASFDNFKFVCDNIKFADDLNQLTGYKKPASRELKVTFFPDLNGDVVAIDYKHYTPSFK 1980

QIH55230.1 ------------------------------------------------------------ 107

YP_009724389.1 KGAKLLHKPIVWHVNNATNKATYKPNTWCIRCLWSTKPVETSNSFDVLKSEDAQGMDNLA 2040

QIH55230.1 ------------------------------------------------------------ 107

YP_009724389.1 CEDLKPVSEEVVENPTIQKDVLECNVKTTEVVGDIILKPANNSLKITEEVGHTDLMAAYV 2100

QIH55230.1 ------------------------------------------------------------ 107

YP_009724389.1 DNSSLTIKKPNELSRVLGLKTLATHGLAAVNSVPWDTIANYAKPFLNKVVSTTTNIVTRC 2160

QIH55230.1 ------------------------------------------------------------ 107

YP_009724389.1 LNRVCTNYMPYFFTLLLQLCTFTRSTNSRIKASMPTTIAKNTVKSVGKFCLEASFNYLKS 2220

QIH55230.1 ------------------------------------------------------------ 107

YP_009724389.1 PNFSKLINIIIWFLLLSVCLGSLIYSTAALGVLMSNLGMPSYCTGYREGYLNSTNVTIAT 2280

QIH55230.1 ------------------------------------------------------------ 107

YP_009724389.1 YCTGSIPCSVCLSGLDSLDTYPSLETIQITISSFKWDLTAFGLVAEWFLAYILFTRFFYV 2340

QIH55230.1 ------------------------------------------------------------ 107

YP_009724389.1 LGLAAIMQLFFSYFAVHFISNSWLMWLIINLVQMAPISAMVRMYIFFASFYYVWKSYVHV 2400

QIH55230.1 ------------------------------------------------------------ 107

YP_009724389.1 VDGCNSSTCMMCYKRNRATRVECTTIVNGVRRSFYVYANGGKGFCKLHNWNCVNCDTFCA 2460

QIH55230.1 ------------------------------------------------------------ 107

YP_009724389.1 GSTFISDEVARDLSLQFKRPINPTDQSSYIVDSVTVKNGSIHLYFDKAGQKTYERHSLSH 2520

QIH55230.1 ------------------------------------------------------------ 107

YP_009724389.1 FVNLDNLRANNTKGSLPINVIVFDGKSKCEESSAKSASVYYSQLMCQPILLLDQALVSDV 2580

QIH55230.1 ------------------------------------------------------------ 107

YP_009724389.1 GDSAEVAVKMFDAYVNTFSSTFNVPMEKLKTLVATAEAELAKNVSLDNVLSTFISAARQG 2640

QIH55230.1 ------------------------------------------------------------ 107

YP_009724389.1 FVDSDVETKDVVECLKLSHQSDIEVTGDSCNNYMLTYNKVENMTPRDLGACIDCSARHIN 2700

QIH55230.1 ------------------------------------------------------------ 107

YP_009724389.1 AQVAKSHNIALIWNVKDFMSLSEQLRKQIRSAAKKNNLPFKLTCATTRQVVNVVTTKIAL 2760

QIH55230.1 ------------------------------------------------------------ 107

YP_009724389.1 KGGKIVNNWLKQLIKVTLVFLFVAAIFYLITPVHVMSKHTDFSSEIIGYKAIDGGVTRDI 2820

QIH55230.1 ------------------------------------------------------------ 107

YP_009724389.1 ASTDTCFANKHADFDTWFSQRGGSYTNDKACPLIAAVITREVGFVVPGLPGTILRTTNGD 2880

QIH55230.1 ------------------------------------------------------------ 107

YP_009724389.1 FLHFLPRVFSAVGNICYTPSKLIEYTDFATSACVLAAECTIFKDASGKPVPYCYDTNVLE 2940

QIH55230.1 ------------------------------------------------------------ 107

YP_009724389.1 GSVAYESLRPDTRYVLMDGSIIQFPNTYLEGSVRVVTTFDSEYCRHGTCERSEAGVCVST 3000

QIH55230.1 ------------------------------------------------------------ 107

YP_009724389.1 SGRWVLNNDYYRSLPGVFCGVDAVNLLTNMFTPLIQPIGALDISASIVAGGIVAIVVTCL 3060

QIH55230.1 ------------------------------------------------------------ 107

YP_009724389.1 AYYFMRFRRAFGEYSHVVAFNTLLFLMSFTVLCLTPVYSFLPGVYSVIYLYLTFYLTNDV 3120

QIH55230.1 ------------------------------------------------------------ 107

YP_009724389.1 SFLAHIQWMVMFTPLVPFWITIAYIICISTKHFYWFFSNYLKRRVVFNGVSFSTFEEAAL 3180

QIH55230.1 ------------------------------------------------------------ 107

YP_009724389.1 CTFLLNKEMYLKLRSDVLLPLTQYNRYLALYNKYKYFSGAMDTTSYREAACCHLAKALND 3240

QIH55230.1 ------------------------------------------------------------ 107

YP_009724389.1 FSNSGSDVLYQPPQTSITSAVLQSGFRKMAFPSGKVEGCMVQVTCGTTTLNGLWLDDVVY 3300

QIH55230.1 ------------------------------------------------------------ 107

YP_009724389.1 CPRHVICTSEDMLNPNYEDLLIRKSNHNFLVQAGNVQLRVIGHSMQNCVLKLKVDTANPK 3360

QIH55230.1 ------------------------------------------------------------ 107

YP_009724389.1 TPKYKFVRIQPGQTFSVLACYNGSPSGVYQCAMRPNFTIKGSFLNGSCGSVGFNIDYDCV 3420

QIH55230.1 ------------------------------------------------------------ 107

YP_009724389.1 SFCYMHHMELPTGVHAGTDLEGNFYGPFVDRQTAQAAGTDTTITVNVLAWLYAAVINGDR 3480

QIH55230.1 ------------------------------------------------------------ 107

YP_009724389.1 WFLNRFTTTLNDFNLVAMKYNYEPLTQDHVDILGPLSAQTGIAVLDMCASLKELLQNGMN 3540

QIH55230.1 ------------------------------------------------------------ 107

YP_009724389.1 GRTILGSALLEDEFTPFDVVRQCSGVTFQSAVKRTIKGTHHWLLLTILTSLLVLVQSTQW 3600

QIH55230.1 ------------------------------------------------------------ 107

YP_009724389.1 SLFFFLYENAFLPFAMGIIAMSAFAMMFVKHKHAFLCLFLLPSLATVAYFNMVYMPASWV 3660

QIH55230.1 ------------------------------------------------------------ 107

YP_009724389.1 MRIMTWLDMVDTSLSGFKLKDCVMYASAVVLLILMTARTVYDDGARRVWTLMNVLTLVYK 3720

QIH55230.1 ------------------------------------------------------------ 107

YP_009724389.1 VYYGNALDQAISMWALIISVTSNYSGVVTTVMFLARGIVFMCVEYCPIFFITGNTLQCIM 3780

QIH55230.1 ------------------------------------------------------------ 107

YP_009724389.1 LVYCFLGYFCTCYFGLFCLLNRYFRLTLGVYDYLVSTQEFRYMNSQGLLPPKNSIDAFKL 3840

QIH55230.1 ------------------------------------------------------------ 107

YP_009724389.1 NIKLLGVGGKPCIKVATVQSKMSDVKCTSVVLLSVLQQLRVESSSKLWAQCVQLHNDILL 3900

QIH55230.1 ------------------------------------------------------------ 107

YP_009724389.1 AKDTTEAFEKMVSLLSVLLSMQGAVDINKLCEEMLDNRATLQAIASEFSSLPSYAAFATA 3960

QIH55230.1 ------------------------------------------------------------ 107

YP_009724389.1 QEAYEQAVANGDSEVVLKKLKKSLNVAKSEFDRDAAMQRKLEKMADQAMTQMYKQARSED 4020

QIH55230.1 ------------------------------------------------------------ 107

YP_009724389.1 KRAKVTSAMQTMLFTMLRKLDNDALNNIINNARDGCVPLNIIPLTTAAKLMVVIPDYNTY 4080

QIH55230.1 ------------------------------------------------------------ 107

YP_009724389.1 KNTCDGTTFTYASALWEIQQVVDADSKIVQLSEISMDNSPNLAWPLIVTALRANSAVKLQ 4140

QIH55230.1 ------------------------------------------------------------ 107

YP_009724389.1 NNELSPVALRQMSCAAGTTQTACTDDNALAYYNTTKGGRFVLALLSDLQDLKWARFPKSD 4200

QIH55230.1 ------------------------------------------------------------ 107

YP_009724389.1 GTGTIYTELEPPCRFVTDTPKGPKVKYLYFIKGLNNLNRGMVLGSLAATVRLQAGNATEV 4260

QIH55230.1 ------------------------------------------------------------ 107

YP_009724389.1 PANSTVLSFCAFAVDAAKAYKDYLASGGQPITNCVKMLCTHTGTGQAITVTPEANMDQES 4320

QIH55230.1 ------------------------------------------------------------ 107

YP_009724389.1 FGGASCCLYCRCHIDHPNPKGFCDLKGKYVQIPTTCANDPVGFTLKNTVCTVCGMWKGYG 4380

QIH55230.1 ------------------------------------------------------------ 107

YP_009724389.1 CSCDQLREPMLQSADAQSFLNRVCGVSAARLTPCGTGTSTDVVYRAFDIYNDKVAGFAKF 4440

QIH55230.1 ------------------------------------------------------------ 107

YP_009724389.1 LKTNCCRFQEKDEDDNLIDSYFVVKRHTFSNYQHEETIYNLLKDCPAVAKHDFFKFRIDG 4500

QIH55230.1 ------------------------------------------------------------ 107

YP_009724389.1 DMVPHISRQRLTKYTMADLVYALRHFDEGNCDTLKEILVTYNCCDDDYFNKKDWYDFVEN 4560

QIH55230.1 ------------------------------------------------------------ 107

YP_009724389.1 PDILRVYANLGERVRQALLKTVQFCDAMRNAGIVGVLTLDNQDLNGNWYDFGDFIQTTPG 4620

QIH55230.1 ------------------------------------------------------------ 107

YP_009724389.1 SGVPVVDSYYSLLMPILTLTRALTAESHVDTDLTKPYIKWDLLKYDFTEERLKLFDRYFK 4680

QIH55230.1 ------------------------------------------------------------ 107

YP_009724389.1 YWDQTYHPNCVNCLDDRCILHCANFNVLFSTVFPPTSFGPLVRKIFVDGVPFVVSTGYHF 4740

QIH55230.1 ------------------------------------------------------------ 107

YP_009724389.1 RELGVVHNQDVNLHSSRLSFKELLVYAADPAMHAASGNLLLDKRTTCFSVAALTNNVAFQ 4800

QIH55230.1 ------------------------------------------------------------ 107

YP_009724389.1 TVKPGNFNKDFYDFAVSKGFFKEGSSVELKHFFFAQDGNAAISDYDYYRYNLPTMCDIRQ 4860

QIH55230.1 ------------------------------------------------------------ 107

YP_009724389.1 LLFVVEVVDKYFDCYDGGCINANQVIVNNLDKSAGFPFNKWGKARLYYDSMSYEDQDALF 4920

QIH55230.1 ------------------------------------------------------------ 107

YP_009724389.1 AYTKRNVIPTITQMNLKYAISAKNRARTVAGVSICSTMTNRQFHQKLLKSIAATRGATVV 4980

QIH55230.1 ------------------------------------------------------------ 107

YP_009724389.1 IGTSKFYGGWHNMLKTVYSDVENPHLMGWDYPKCDRAMPNMLRIMASLVLARKHTTCCSL 5040

QIH55230.1 ------------------------------------------------------------ 107

YP_009724389.1 SHRFYRLANECAQVLSEMVMCGGSLYVKPGGTSSGDATTAYANSVFNICQAVTANVNALL 5100

QIH55230.1 ------------------------------------------------------------ 107

YP_009724389.1 STDGNKIADKYVRNLQHRLYECLYRNRDVDTDFVNEFYAYLRKHFSMMILSDDAVVCFNS 5160

QIH55230.1 ------------------------------------------------------------ 107

YP_009724389.1 TYASQGLVASIKNFKSVLYYQNNVFMSEAKCWTETDLTKGPHEFCSQHTMLVKQGDDYVY 5220

QIH55230.1 ------------------------------------------------------------ 107

YP_009724389.1 LPYPDPSRILGAGCFVDDIVKTDGTLMIERFVSLAIDAYPLTKHPNQEYADVFHLYLQYI 5280

QIH55230.1 ------------------------------------------------------------ 107

YP_009724389.1 RKLHDELTGHMLDMYSVMLTNDNTSRYWEPEFYEAMYTPHTVLQAVGACVLCNSQTSLRC 5340

QIH55230.1 ------------------------------------------------------------ 107

YP_009724389.1 GACIRRPFLCCKCCYDHVISTSHKLVLSVNPYVCNAPGCDVTDVTQLYLGGMSYYCKSHK 5400

QIH55230.1 ------------------------------------------------------------ 107

YP_009724389.1 PPISFPLCANGQVFGLYKNTCVGSDNVTDFNAIATCDWTNAGDYILANTCTERLKLFAAE 5460

QIH55230.1 ------------------------------------------------------------ 107

YP_009724389.1 TLKATEETFKLSYGIATVREVLSDRELHLSWEVGKPRPPLNRNYVFTGYRVTKNSKVQIG 5520

QIH55230.1 ------------------------------------------------------------ 107

YP_009724389.1 EYTFEKGDYGDAVVYRGTTTYKLNVGDYFVLTSHTVMPLSAPTLVPQEHYVRITGLYPTL 5580

QIH55230.1 ------------------------------------------------------------ 107

YP_009724389.1 NISDEFSSNVANYQKVGMQKYSTLQGPPGTGKSHFAIGLALYYPSARIVYTACSHAAVDA 5640

QIH55230.1 ------------------------------------------------------------ 107

YP_009724389.1 LCEKALKYLPIDKCSRIIPARARVECFDKFKVNSTLEQYVFCTVNALPETTADIVVFDEI 5700

QIH55230.1 ------------------------------------------------------------ 107

YP_009724389.1 SMATNYDLSVVNARLRAKHYVYIGDPAQLPAPRTLLTKGTLEPEYFNSVCRLMKTIGPDM 5760

QIH55230.1 ------------------------------------------------------------ 107

YP_009724389.1 FLGTCRRCPAEIVDTVSALVYDNKLKAHKDKSAQCFKMFYKGVITHDVSSAINRPQIGVV 5820

QIH55230.1 ------------------------------------------------------------ 107

YP_009724389.1 REFLTRNPAWRKAVFISPYNSQNAVASKILGLPTQTVDSSQGSEYDYVIFTQTTETAHSC 5880

QIH55230.1 ------------------------------------------------------------ 107

YP_009724389.1 NVNRFNVAITRAKVGILCIMSDRDLYDKLQFTSLEIPRRNVATLQAENVTGLFKDCSKVI 5940

QIH55230.1 ------------------------------------------------------------ 107

YP_009724389.1 TGLHPTQAPTHLSVDTKFKTEGLCVDIPGIPKDMTYRRLISMMGFKMNYQVNGYPNMFIT 6000

QIH55230.1 ------------------------------------------------------------ 107

YP_009724389.1 REEAIRHVRAWIGFDVEGCHATREAVGTNLPLQLGFSTGVNLVAVPTGYVDTPNNTDFSR 6060

QIH55230.1 ------------------------------------------------------------ 107

YP_009724389.1 VSAKPPPGDQFKHLIPLMYKGLPWNVVRIKIVQMLSDTLKNLSDRVVFVLWAHGFELTSM 6120

QIH55230.1 ------------------------------------------------------------ 107

YP_009724389.1 KYFVKIGPERTCCLCDRRATCFSTASDTYACWHHSIGFDYVYNPFMIDVQQWGFTGNLQS 6180

QIH55230.1 ------------------------------------------------------------ 107

YP_009724389.1 NHDLYCQVHGNAHVASCDAIMTRCLAVHECFVKRVDWTIEYPIIGDELKINAACRKVQHM 6240

QIH55230.1 ------------------------------------------------------------ 107

YP_009724389.1 VVKAALLADKFPVLHDIGNPKAIKCVPQADVEWKFYDAQPCSDKAYKIEELFYSYATHSD 6300

QIH55230.1 ------------------------------------------------------------ 107

YP_009724389.1 KFTDGVCLFWNCNVDRYPANSIVCRFDTRVLSNLNLPGCDGGSLYVNKHAFHTPAFDKSA 6360

QIH55230.1 ------------------------------------------------------------ 107

YP_009724389.1 FVNLKQLPFFYYSDSPCESHGKQVVSDIDYVPLKSATCITRCNLGGAVCRHHANEYRLYL 6420

QIH55230.1 ------------------------------------------------------------ 107

YP_009724389.1 DAYNMMISAGFSLWVYKQFDTYNLWNTFTRLQSLENVAFNVVNKGHFDGQQGEVPVSIIN 6480

QIH55230.1 ------------------------------------------------------------ 107

YP_009724389.1 NTVYTKVDGVDVELFENKTTLPVNVAFELWAKRNIKPVPEVKILNNLGVDIAANTVIWDY 6540

QIH55230.1 ------------------------------------------------------------ 107

YP_009724389.1 KRDAPAHISTIGVCSMTDIAKKPTETICAPLTVFFDGRVDGQVDLFRNARNGVLITEGSV 6600

QIH55230.1 ------------------------------------------------------------ 107

YP_009724389.1 KGLQPSVGPKQASLNGVTLIGEAVKTQFNYYKKVDGVVQQLPETYFTQSRNLQEFKPRSQ 6660

QIH55230.1 ------------------------------------------------------------ 107

YP_009724389.1 MEIDFLELAMDEFIERYKLEGYAFEHIVYGDFSHSQLGGLHLLIGLAKRFKESPFELEDF 6720

QIH55230.1 ------------------------------------------------------------ 107

YP_009724389.1 IPMDSTVKNYFITDAQTGSSKCVCSVIDLLLDDFVEIIKSQDLSVVSKVVKVTIDYTEIS 6780

QIH55230.1 ------------------------------------------------------------ 107

YP_009724389.1 FMLWCKDGHVETFYPKLQSSQAWQPGVAMPNLYKMQRMLLEKCDLQNYGDSATLPKGIMM 6840

QIH55230.1 ------------------------------------------------------------ 107

YP_009724389.1 NVAKYTQLCQYLNTLTLAVPYNMRVIHFGAGSDKGVAPGTAVLRQWLPTGTLLVDSDLND 6900

QIH55230.1 ------------------------------------------------------------ 107

YP_009724389.1 FVSDADSTLIGDCATVHTANKWDLIISDMYDPKTKNVTKENDSKEGFFTYICGFIQQKLA 6960

QIH55230.1 ------------------------------------------------------------ 107

YP_009724389.1 LGGSVAIKITEHSWNADLYKLMGHFAWWTAFVTNVNASSSEAFLIGCNYLGKPREQIDGY 7020

QIH55230.1 ------------------------------------------------------------ 107

YP_009724389.1 VMHANYIFWRNTNPIQLSSYSLFDMSKFPLKLRGTAVMSLKEGQINDMILSLLSKGRLII 7080

QIH55230.1 ---------------- 107

YP_009724389.1 RENNRVVISSDVLVNN 7096
